# Selenoprotein I is indispensable for ether lipid homeostasis and proper myelination

**DOI:** 10.1016/j.jbc.2024.107259

**Published:** 2024-04-04

**Authors:** Lance G.A. Nunes, Chi Ma, FuKun W. Hoffmann, Ashley E. Shay, Matthew W. Pitts, Peter R. Hoffmann

**Affiliations:** 1 Department of Anatomy, Biochemistry, and Physiology, John A. Burns School of Medicine, University of Hawaii, Honolulu, Hawaii, USA; 2 Department of Cell and Molecular Biology, John A. Burns School of Medicine, University of Hawaii, Honolulu, Hawaii, USA; 3 Huck Institutes of the Life Sciences, The Pennsylvania State University, University Park, Pennsylvania, USA; 4 Department of Veterinary and Biomedical Sciences, The Pennsylvania State University, University Park, Pennsylvania, USA

**Keywords:** selenoprotein, myelin, ether lipid, lipid peroxidation, hereditary spastic paraplegia, oligodendrocyte, corticospinal tract

## Abstract

Selenoprotein I (SELENOI) catalyzes the final reaction of the CDP-ethanolamine branch of the Kennedy pathway, generating the phospholipids phosphatidylethanolamine (PE) and plasmenyl-PE. Plasmenyl-PE is a key component of myelin and is characterized by a vinyl ether bond that preferentially reacts with oxidants, thus serves as a sacrificial antioxidant. In humans, multiple loss-of-function mutations in genes affecting plasmenyl-PE metabolism have been implicated in hereditary spastic paraplegia, including SELENOI. Herein, we developed a mouse model of nervous system-restricted SELENOI deficiency that circumvents embryonic lethality caused by constitutive deletion and recapitulates phenotypic features of hereditary spastic paraplegia. Resulting mice exhibited pronounced alterations in brain lipid composition, which coincided with motor deficits and neuropathology including hypomyelination, elevated reactive gliosis, and microcephaly. Further studies revealed increased lipid peroxidation in oligodendrocyte lineage cells and disrupted oligodendrocyte maturation both *in vivo* and *in vitro*. Altogether, these findings detail a critical role for SELENOI-derived plasmenyl-PE in myelination that is of paramount importance for neurodevelopment.

Cells rely on integrated biosynthetic pathways to maintain balanced lipid composition in membranes while enzymatic and nonenzymatic mechanisms protect these lipids from oxidative damage. The most abundant membrane phospholipids are phosphatidylcholine (PC) and phosphatidylethanolamine (PE), which are synthesized in the endoplasmic reticulum through the choline and ethanolamine branches of the Kennedy pathway, respectively (1, 2). While most PC and PE species are diacylated at the *sn-1* and *-2* positions, a substantial fraction contain ether bonds at the *sn-1* position of the glycerol backbone. The central nervous system (CNS) is enriched in plasmenyl-PE, a subclass of PE species characterized by a vinyl-ether bond at the *sn*-1 position. Due to the labile nature of the vinyl-ether bond, plasmenyl-PE acts as an antioxidant that is preferentially oxidized over polyunsaturated fatty acids, thereby protecting against lipid peroxidation (3, 4, 5). Despite the significance of this protective mechanism for preserving cellular viability and function, limited data exists from *in vivo* models investigating the role of plasmenyl-PE in brain.

Synthesis of diacyl-PE and plasmenyl-PE relies on the ethanolamine branch of the Kennedy pathway, of which selenoprotein I (SELENOI) conducts the terminal reaction. SELENOI belongs to two distinct protein families: (1) lipid phosphotransferases, defined by a conserved cytidine diphosphate-alcohol phosphotransferase motif, and (2) selenoproteins, characterized by a selenocysteine residue that requires a unique translational process for incorporation (6). This enzyme serves to transfer the ethanolamine phosphate group from CDP-ethanolamine to one of two lipid donors, 1,2-diacylglycerol or 1-alkyl-2-acylglycerol (AAG), to generate diacyl-PE or plasmanyl-PE, respectively (7). Plasmanyl-PE is subsequently converted to plasmenyl-PE *via* desaturation of the 1-alkyl group to a 1-alkenyl group by plasmanylethanolamine desaturase, recently identified as TMEM189 (8). Thus, the end products of SELENOI-dependent pathways are diacyl-PE and plasmenyl-PE, with individual species identified based on length and degree of saturation at *sn-1* and *-2* positions.

While SELENOI is indispensable for murine embryonic development (9), rare loss-of-function mutations in humans lead to a form of hereditary spastic paraplegia (HSP) characterized by motor impairment, microcephaly, and hypomyelination (10, 11, 12). Due to nervous system inaccessibility in patients, human studies have been limited in their ability to directly measure lipid composition in the brain. Cellular models have been informative, as SELENOI deletion in HeLa cells reduced plasmenyl-PE species to a greater degree than diacyl-PE species (11). This finding was corroborated in patient-derived fibroblasts and HEK293 cells with SELENOI loss-of-function mutations (11, 13), as well as our murine *ex vivo* T cell studies (14).

Given the prospective importance of SELENOI to the brain, we developed a mouse model where SELENOI deficiency is restricted to the nervous system. This was achieved using a transgenic strain previously used for brain–specific KO studies (15, 16, 17, 18, 19), where Cre recombinase is driven by the tubulin-1α promoter (*Tuba1a*-*Cre*). Resulting mice (*Tuba1a-Cre::SELENOI*^*fl/fl*^) exhibited striking alterations in the brain ether lipid composition, which coincided with severe motor deficits and neuropathology including hypomyelination, elevated reactive gliosis, and microcephaly. Additional studies utilizing flow cytometry and primary cortical cultures determined that SELENOI deficiency leads to a reduction in mature oligodendrocytes, along with increased lipid peroxidation. In summary, these results illustrate the vital function of SELENOI in neurodevelopment.

## Results

### Validation of Tuba1a-Cre::SELENOI^fl/fl^ mouse model

**Figure 1:**
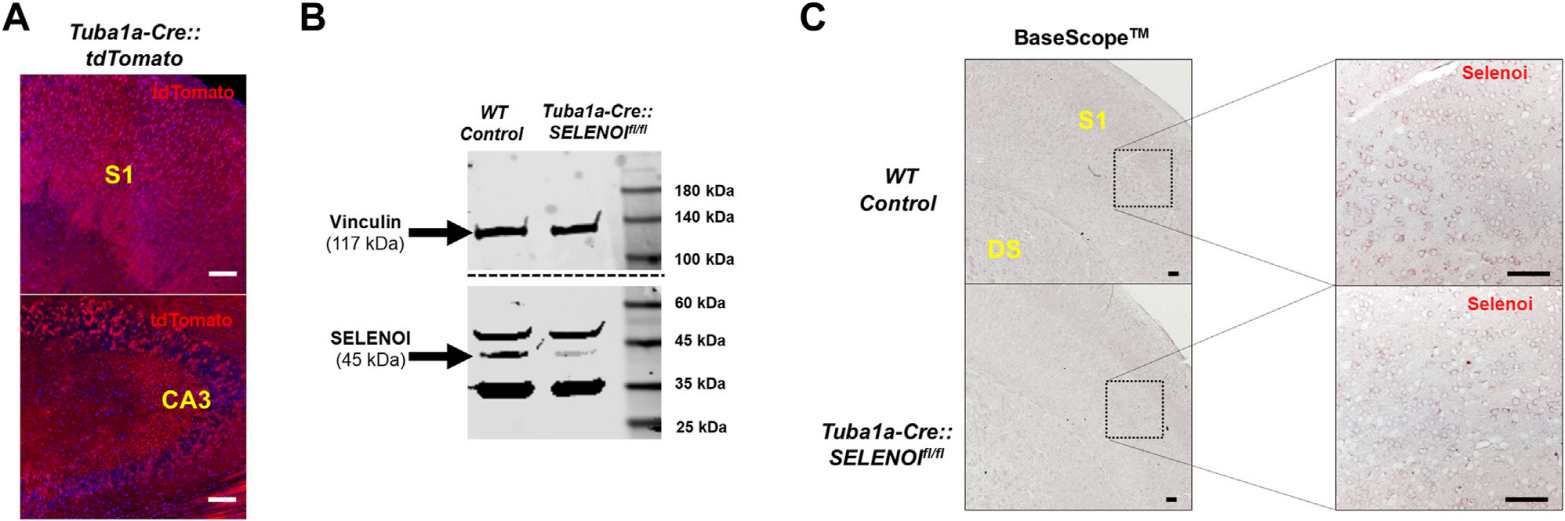
**Validation of *Tuba1a-Cre::SELENOI***^***fl/fl***^**mouse model.***A*, confocal images of tdTomato labeling in the somatosensory cortex (S1) and hippocampal CA3 region of *Tuba1a-Cre::ROSA26R*^tdTomato^ reporter mice. *B*, Western blot verifying reduced protein levels of SELENOI in whole brain samples of *Tuba1a-Cre::SELENOI*^*fl/fl*^ mice. *C*, images of BaseScope chromogenic labeling of SELENOI mRNA in S1. Note reduced levels in *Tuba1a-Cre::SELENOI*^*fl/fl*^ mice. The scale bar represents 100 μm. SELENOI, selenoprotein I.

To profile the extent of Cre-driven recombination elicited in our mouse model, we interbred *Tuba1a-Cre*^*+/−*^ and *ROSA26R*^**tdTomato**^ mice. As anticipated, tdTomato expression was widespread throughout the mouse brain (Fig. 1*A*). Western blot analyses confirmed that SELENOI protein levels were markedly reduced in whole brain samples of *Tuba1a-Cre::SELENOI*^*fl/fl*^ mice (Fig. 1*B*). Likewise, quantitative PCR analyses showed that SELENOI mRNA levels were significantly diminished in the brain, but not muscle, of *Tuba1a-Cre::SELENOI*^*fl/fl*^ mice relative to controls (Fig. S1). Further studies using the chromogenic BaseScope *in situ* system verified that SELENOI mRNA was largely absent in the brain of *Tuba1a-Cre::SELENOI*^*fl/fl*^ mice (Fig. 1*C*). Moreover, in WT control brains, SELENOI mRNA was widespread, in line with publicly available data compiled at the Human Protein Atlas (20) detailing broad expression of SELENOI across brain regions and cell types.

### Phenotypic characterization of Tuba1a-Cre::SELENOI^fl/fl^ mice

**Figure 2:**
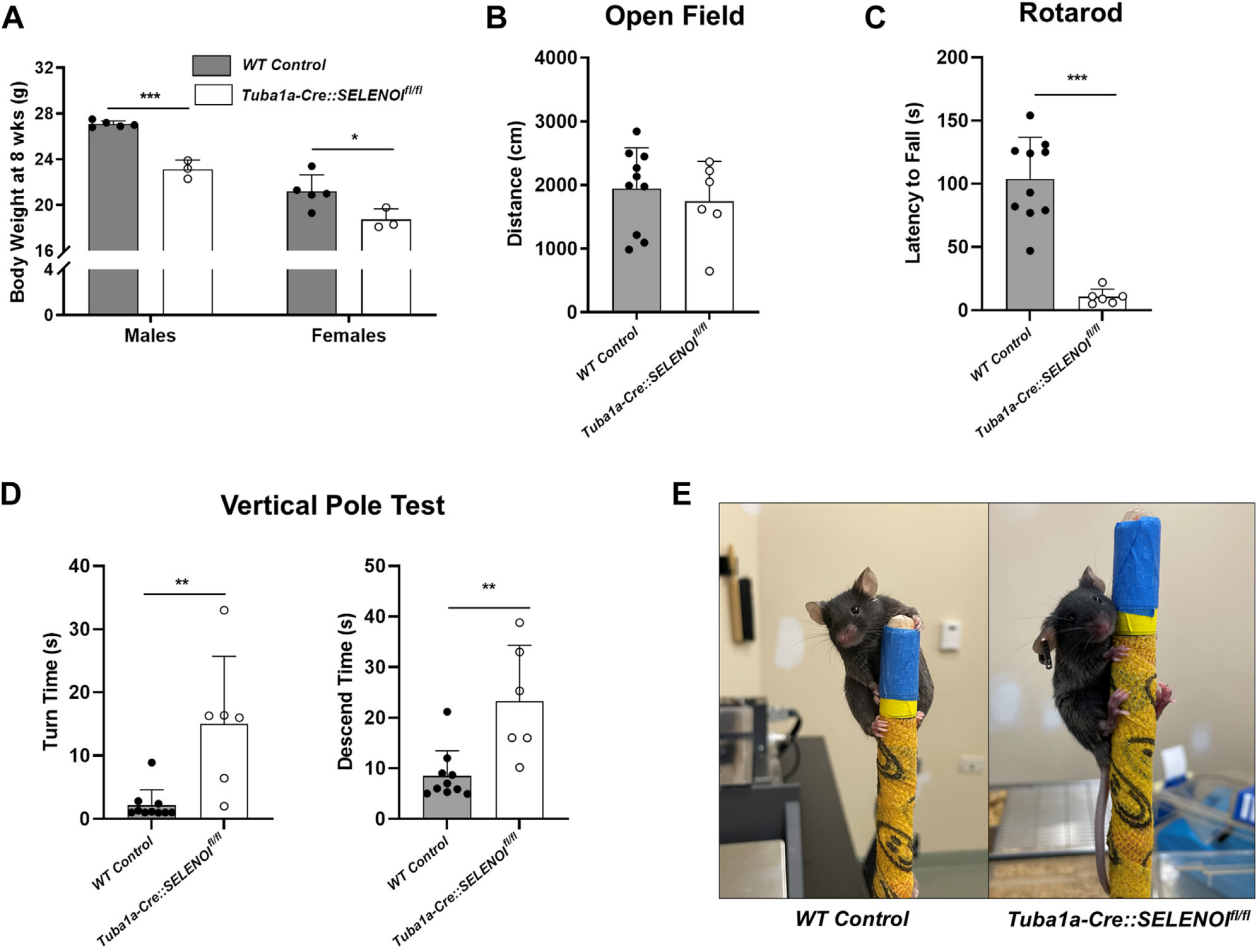
**Characterization of *Tuba1a-Cre::SELENOI***^***fl/fl***^**mice.***A*, body weights for male and female WT and *Tuba1a-Cre::SELENOI*^*fl/fl*^ mice from 8 to 12 weeks of age. *B*, distance traveled during the open field test (5 min). *C*, latency to fall off the Rotarod. *D*, time to turn downward and descend the vertical pole. *E*, image of *Tuba1a-Cre::SELENOI*^*fl/fl*^ mouse during the vertical pole test. Note the inability to grasp pole with hindlimbs. N = 6 to 10 mice per genotype. In all plots, points represent measurements from a single mouse and bars represent mean ± SD. ∗*p* < 0.05, ∗∗*p* < 0.01, ∗∗∗*p* < 0.001. SELENOI, selenoprotein I.

We next assessed the phenotypic effects of nervous system–specific KO of SELENOI in young adult mice aged 8 to 12 weeks. Body weights of male (*t*_*6*_ = 10.48, *p* < 0.0001) and female (*t*_*6*_ = 2.555, *p* = 0.0432) *Tuba1a-Cre::SELENOI*^*fl/fl*^ mice were significantly lower at 8 weeks (Fig. 2*A*), which parallels reports of reduced growth in humans with SELENOI mutations (10, 11). When subjected to the open field test, locomotion was comparable between groups, indicating that *Tuba1a-Cre::SELENOI*^*fl/fl*^ mice can ambulate relatively normally (Fig. 2*B*). Yet, on the rotarod test of motor coordination, mice exhibited profound impairment, immediately falling off the rod once it started rotating (*t*_*14*_ = 6.766, *p* < 0.0001) (Figs. 2*C* and S2). Likewise, similar deficits were apparent in the vertical pole test, an assay used to assess coordination and basal ganglia-related movement disorders (Figs. 2*D* and S2) (21, 22, 23). For this assay, rodents are placed atop a vertical pole and the time for the animal to orient downward (turn) and descend the length of the pole are measured. *Tuba1a-Cre::SELENOI*^*fl/fl*^ mice took significantly longer to orient downward (*t*_*14*_ = 3.731, *p* = 0.0022) and descend the pole (*t*_*14*_ = 3.682, *p* = 0.0025). Additionally, several *Tuba1a-Cre::SELENOI*^*fl/fl*^ mice were unable to grasp the vertical pole with their hindlimbs (Fig. 2*E*).

### SELENOI deficiency in the CNS results in reactive gliosis, deficient myelination, and microcephaly

**Figure 3:**
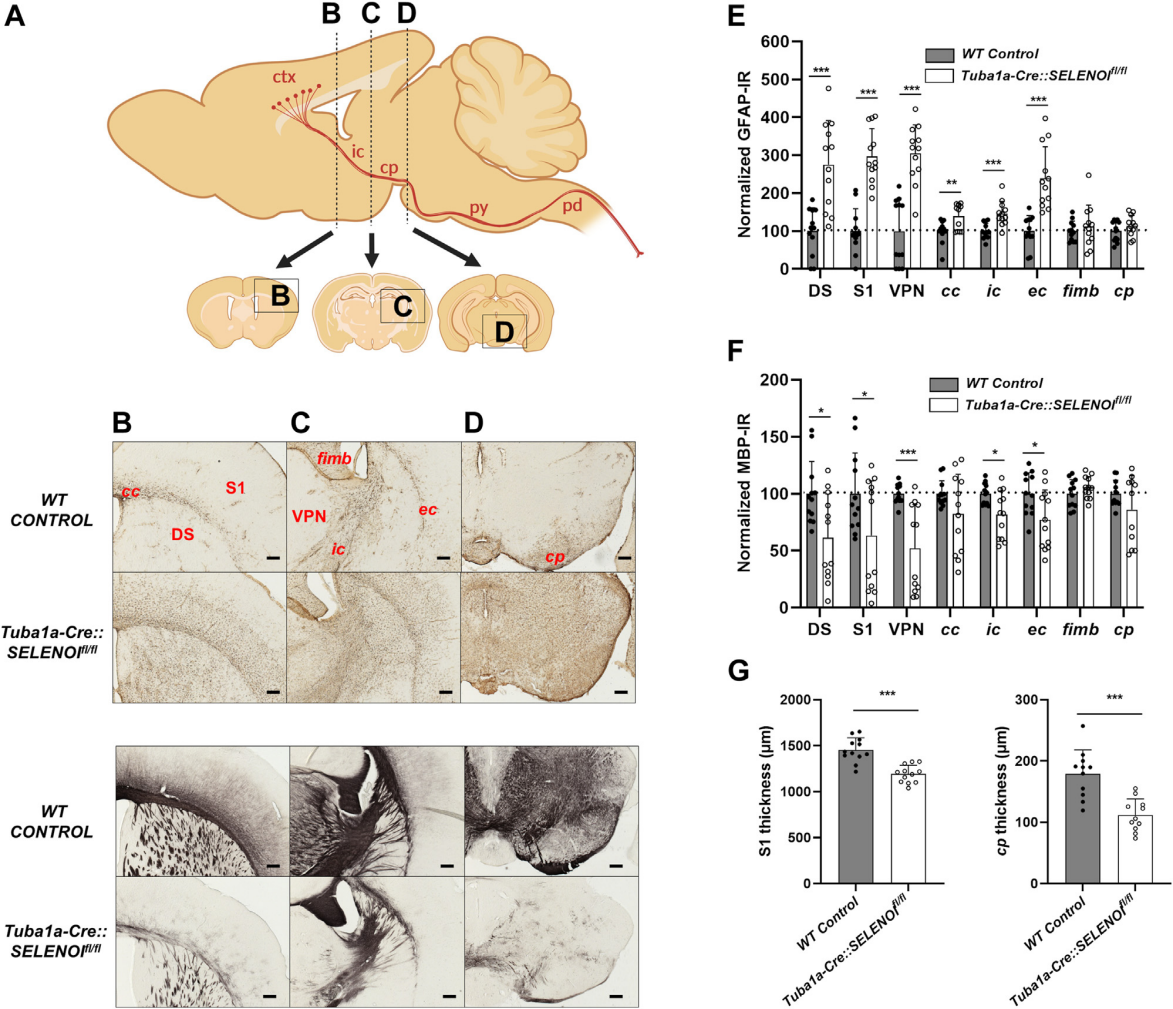
**Neuropathology of *Tuba1a-Cre::SELENOI***^***fl/fl***^**mice.***A*, sagittal diagram of the mouse brain detailing the corticospinal tract in *red*. *Dotted lines* correspond to coronal sections used for image analysis. *B*–*D*, images of GFAP (*top*) and MBP (*bottom*) immunostained sections corresponding to the somatosensory cortex (*B*), internal capsule (*C*), and the midbrain (*D*). *E*, quantification of GFAP-IR in specific brain regions. *F*, quantification of MBP-IR in specific brain regions *G*, measured thickness of the somatosensory cortex (*left*) and cerebral peduncle (*right*). N = 12 images from six mice per genotype for each brain region. In all plots, *points* represent measurements from a single image and *bars* represent mean ± SD. The scale bar represents 100 μm. ∗*p* < 0.05, ∗∗*p* < 0.01, ∗∗∗*p* < 0.001. GFAP, glial fibrillary acidic protein; MBP, myelin basic protein; SELENOI, selenoprotein I.

**Figure 4:**
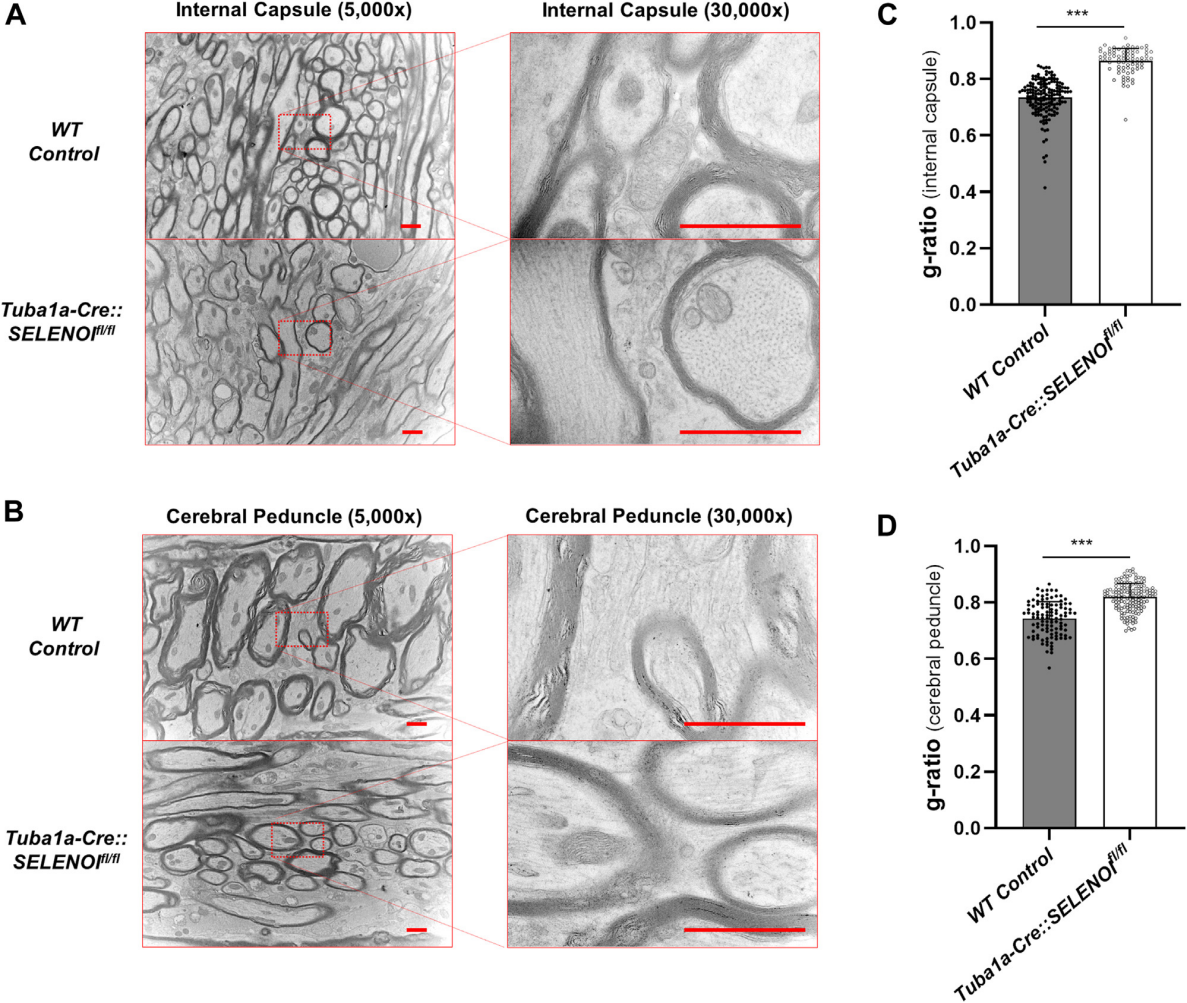
**Myelin defects in the corticospinal tract of *Tuba1a-Cre::SELENOI***^***fl/fl***^**mice.***A* and *B*, transmission electron microscopy images of myelin in the internal capsule (*A*) and cerebral peduncle (*B*). *C* and *D*, quantification of myelin by determination of the g-ratio in the internal capsule (*C*), and cerebral peduncle (*D*). In all plots, points represent measurements from a single image and *bars* represent mean ± SD. The scale bar represents 1 μm. ∗*p* < 0.05, ∗∗∗*p* < 0.001. SELENOI, selenoprotein I.

Following behavioral testing, extensive histological analyses were conducted to characterize underlying neuropathological alterations. Upregulation of glial fibrillary acidic protein (GFAP), indicative of reactive gliosis, was evident throughout the brains of *Tuba1a-Cre::SELENOI*^*fl/fl*^ mice (Fig. 3, *A*–*E*). Prominent increases were observed in the dorsal striatum (*t*_*22*_ = 7.202, *p* < 0.0001), primary somatosensory cortex (S1) (*t*_*22*_ = 8.114, *p* < 0.0001), and the ventral posterior nucleus of the thalamus (*t*_*22*_ = 8.417, *p* < 0.0001), all regions implicated in motor control. GFAP levels were also elevated in white matter, albeit to a lesser extent, with significant increases in the corpus callosum (*cc*) (*t*_*22*_ = 3.054, *p* = 0.0058), internal capsule (*ic*) (*t*_*22*_ = 4.168, *p* = 0.0004), and external capsule (*ec*) (*t*_*22*_ = 5.230, *p* < 0.0001). Conversely, striking reductions in levels of myelin basic protein (MBP) occurred in regions displaying GFAP upregulation, including dorsal striatum (*t*_*22*_ = 2.807, *p* = 0.0103), S1 (*t*_*22*_ = 2.128, *p* = 0.0448), ventral posterior nucleus of the thalamus (*t*_*22*_ = 4.213, *p* = 0.0004), and *ic* (*t*_*22*_ = 2.776, *p* = 0.0110) (Fig. 3, *A*–*F*). Notably, MBP levels were normal in many nonmotor tracts, including the fimbria, optic tract, and stria terminalis (Fig. S3). We also examined MBP expression in the sciatic nerve of the peripheral nervous system and observed no distinct qualitative alterations (Fig. S3), suggesting that motor deficits were largely a result of CNS impairment. Brains of *Tuba1a-Cre::SELENOI*^*fl/fl*^ mice were also discernibly smaller, as the measured thickness of S1 (*t*_*22*_ = 5.560, *p* < 0.0001) and *cp* (*t*_*22*_ = 5.077, *p* < 0.0001) were reduced to roughly 80% and 60% of WT controls, respectively (Fig. 3*G*). In addition, degenerating neurons were detected *via* silver staining along the corticospinal tract at multiple coronal levels, with staining most prevalent in the *ic* (Fig. S4). We also measured the density of parvalbumin-expressing interneurons (PVIs) in the S1 region of the cortex. PVIs are a highly metabolic class of GABAergic interneurons that are especially vulnerable to oxidative stress (24, 25, 26) and ferroptosis (27). Surprisingly, we observed no differences in PVI density between genotypes, albeit there was a slight, nonsignificant trend upward in *Tuba1a-Cre::SELENOI*^*fl/fl*^ mice that may be reflective of reduced brain size (Fig. S5). Further studies using electron microscopy examined the fine structure of the myelin sheath along the corticospinal tract, in sections containing the *ic* (Fig. 4*A*) or the cerebral peduncle (*cp*) (Fig. 4*B*). Myelin was conspicuously reduced, as measured g-ratios were significantly increased for *Tuba1a-Cre::SELENOI*^*fl/fl*^ mice by 18% in the *ic* (*t*_*244*_ = 16.06, *p* < 0.0001) and 10% in the *cp* (*t*_*259*_ = 11.47, *p* < 0.0001) (Fig. 4, *C* and *D*). Additional analyses revealed that myelination of the sciatic nerves of *Tuba1a-Cre::SELENOI*^*fl/fl*^ mice were affected to a lesser degree, with mean g-ratios increased by 5% relative to WT controls (Fig. S6).

### SELENOI deficiency alters composition of ether lipids in the brain

**Figure 5:**
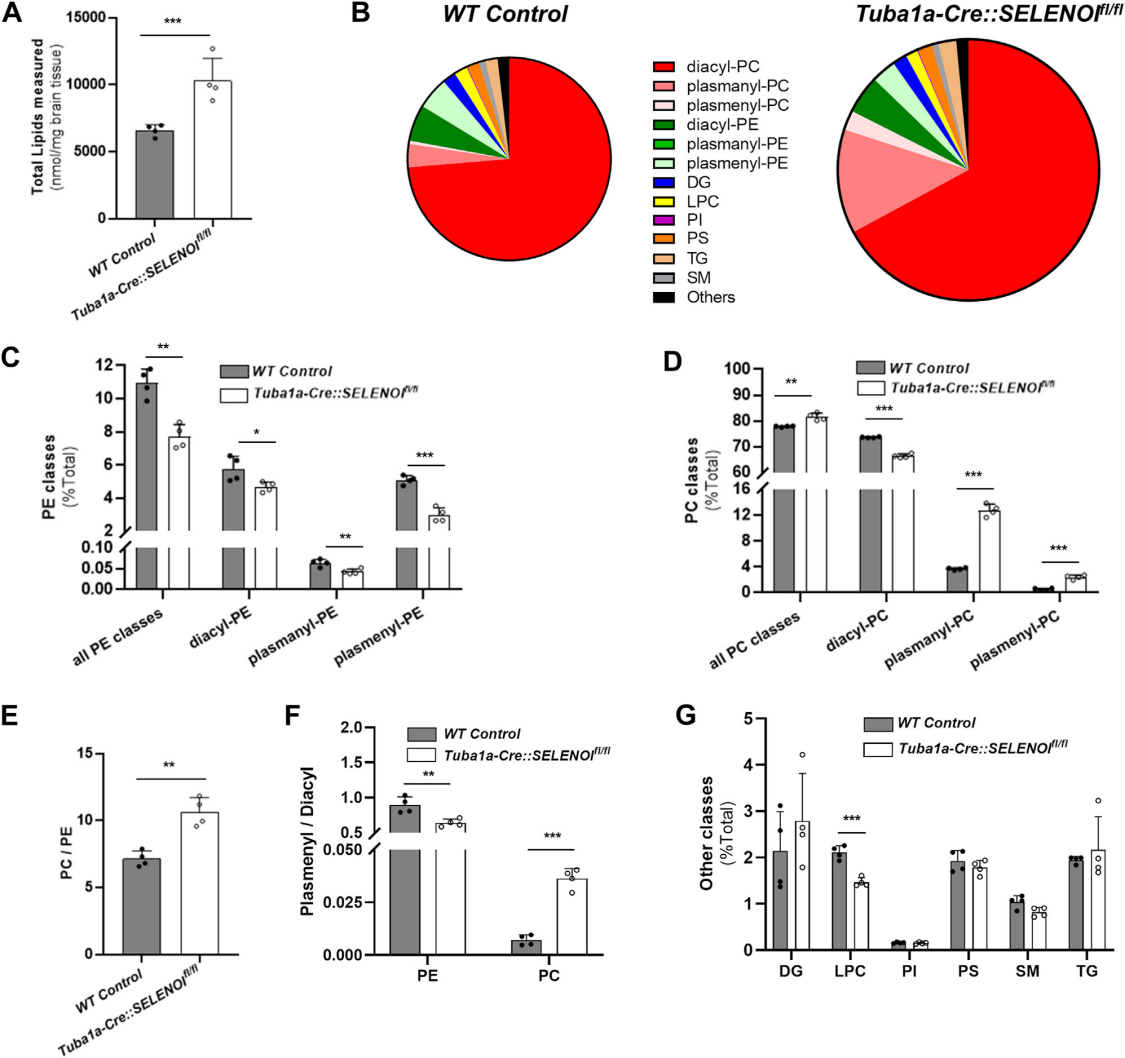
**SELENOI deficiency alters composition of ether lipids in brain.***A*, total lipids measured (nmol) per mg of brain tissue. *B*, pie chart diagram detailing relative abundance of major lipid classes. Note that the pie for *Tuba1a-Cre::SELENOI*^*fl/fl*^ is proportionally larger to represent increased total lipids. *C*, relative abundance of main PE lipid classes. *D*, relative abundance of main PC lipid classes. *E*, ratio of total PC to total PE for all lipid classes. *F*, ratio of plasmenyl to diacyl lipid classes for PE and PC. *G*, relative abundance of major lipid classes synthesized outside of the Kennedy pathway. N = 4 brains per genotype. In all plots, *points* represent measurements from a single mouse and *bars* represent mean ± SD. ∗*p* < 0.05, ∗∗*p* < 0.01, ∗∗∗*p* < 0.001. SELENOI, selenoprotein I.

Our next set of experiments exploited nontargeted, unbiased lipidomics to compare the brain lipid composition in WT and *Tuba1a-Cre::SELENOI*^*fl/fl*^ mice at 3 weeks of age, which corresponds to the most rapid phase of myelination (28, 29) (accession number MSV000094464). Measurements were made for 1095 individual lipid species from 46 distinct lipid classes, including those synthesized through the ethanolamine and choline branches of the Kennedy pathway. Interestingly, when normalized for weight, significantly higher concentrations of total lipid species were observed in *Tuba1a-Cre::SELENOI*^*fl/fl*^ brains (*t*_*6*_ = 4.336, *p* = 0.0049) (Fig. 5*A*).The most prevalent lipid classes were grouped and displayed in pie-chart format to illustrate their relative abundance (Fig. 5*B*). Classes included diacylated phospholipids (diacyl-PC, diacyl-PE, phosphatidylinositol, and phosphatidylserine), alkyl ether phospholipids (plasmanyl-PC, plasmanyl-PE), vinyl ether phospholipids (plasmenyl-PC, -PE), diglycerides, lysophosphatidylcholine, sphingomyelin, triglycerides, and others. When relative abundance was compared, differences were observed in all lipid classes synthesized by the Kennedy pathways. *Tuba1a-Cre::SELENOI*^*fl/fl*^ mice exhibited decreased levels of all PE classes (All PE: *t*_*6*_ = 5.742, *p* = 0.0012; diacyl-PE: *t*_*6*_ = 2.721, *p* = 0.0346; plasmanyl-PE: *t*_*6*_ 4.008, *p* = 0.0071), with most robust reductions observed for plasmenyl-PE (*t*_*6*_ = 8.309, *p* = 0.0002) (Fig. 5*C*). In contrast, levels of ether-linked PC classes (plasmanyl-PC: *t*_*6*_ = 19.17, *p* < 0.0001; plasmenyl-PC: *t*_*6*_ = 10.10, *p* < 0.0001) were dramatically increased (Fig. 5*D*), likely to compensate for diminished plasmenyl-PE. This resulted in elevated levels of total PC species in *Tuba1a-Cre::SELENOI*^*fl/fl*^ mice (*t*_*6*_ = 5.186, *p* = 0.0020), even though levels of diacyl-PC were significantly lower (*t*_*6*_ = 17.98, *p* < 0.0001). Additionally, these alterations led to a significant increase in the ratio of PC to PE (*t*_*6*_ = 4.336, *p* = 0.0049) (Fig. 5*E*), changes that could influence membrane dynamics and fluidity (30). Moreover, we also found that SELENOI deficiency distorted the ratiometric balance of plasmenyl to diacyl species for both PE (*t*_*6*_ = 3.978, *p* = 0.0073) and PC (*t*_*6*_ = 10.91, *p* < 0.0001) (Fig. 5*F*). Further examination of lipidomic data for PE and PC identified individual lipid species most affected by SELENOI deficiency and also revealed that lipids containing docosahexaenoic acid (22:6) tended to be the most dysregulated (Fig. S7). Finally, analyses of lipid classes synthesized outside of the Kennedy pathways showed comparable levels between groups (Fig. 5*G*), with the exception of lysophosphatidylcholine (*t*_*6*_ = 7.274, *p* = 0.0003), which was reduced in *Tuba1a-Cre::SELENOI*^*fl/fl*^ mice.

### Increased lipid peroxidation and reduced numbers of mature oligodendrocytes in Tuba1a-Cre::SELENOI^fl/fl^ mice

**Figure 6:**
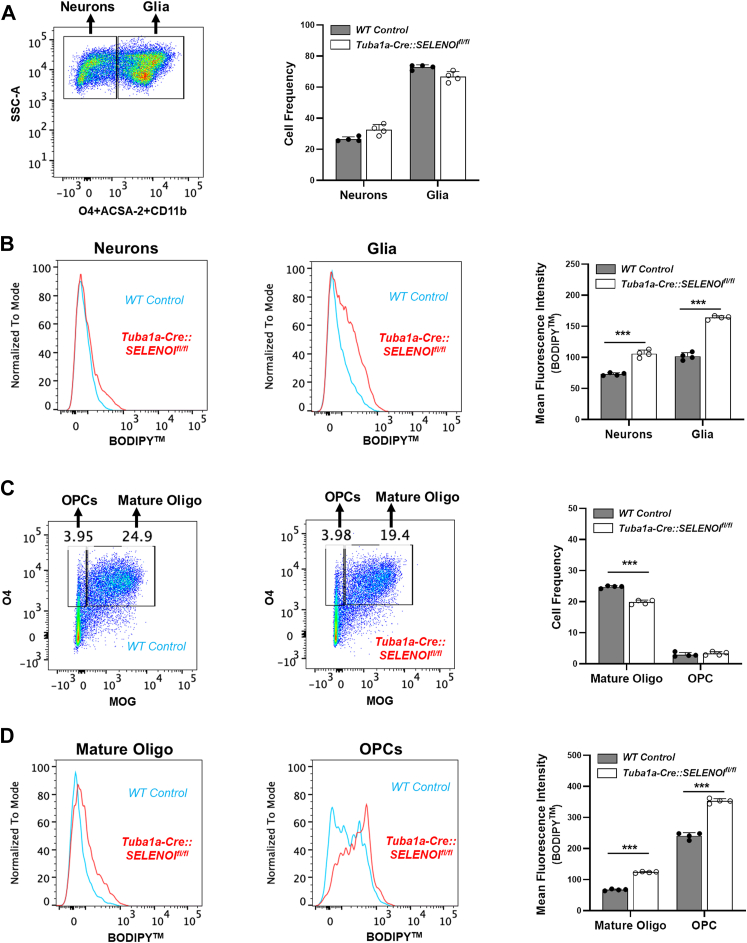
**SELENOI deficiency leads to a reduction in mature oligodendrocytes and increased lipid peroxidation.***A*, flow cytometry dot plots depicting the gating strategy used to distinguish neurons from glia (*left*) and resulting observed frequencies of neurons and glia (*right*). *B*, representative histograms for BODIPY fluorescence in neurons and glia along with corresponding bar graphs for mean fluorescence intensity. *C*, dot plots depicting the gating strategy used to distinguish mature oligodendrocytes from OPCs (*left*) and resulting observed frequencies of mature oligodendrocytes and OPCs (*right*). *D*, representative histograms for BODIPY fluorescence in mature oligodendrocytes and OPCs along with corresponding bar graphs for mean fluorescence intensity. ∗∗∗*p* < 0.001. OPC, oligodendrocyte precursor cell; SELENOI, selenoprotein I.

Given the presumed antioxidant function of plasmenyl-PE, we next investigated whether the observed reduction in *Tuba1a-Cre::SELENOI*^*fl/fl*^ mice leads to increased lipid peroxidation. To interrogate this question, we used a flow cytometry approach to measure lipid peroxidation in various neural cell types of the developing mouse brain. Dissociated brain cells from 3 week-old mice were labeled with the lipid peroxidation sensor, BODIPY, along with cell-type specific markers for astrocytes (ACSA-2), microglia (CD11b), and oligodendrocytes (O4). Cells negative for all markers were classified as neurons. The relative frequency of neurons and glial cells were comparable between genotypes, but lipid peroxidation levels were significantly elevated in both neurons (*t*_*6*_ = 10.57, *p* < 0.0001) and glia (*t*_*6*_ = 19.17, *p* < 0.0001) of *Tuba1a-Cre::SELENOI*^*fl/fl*^ mice (Fig. 6, *A* and *B*). Further analyses were conducted using an antibody targeting the myelin oligodendrocyte glycoprotein to distinguish oligodendrocyte precursor cells (OPCs) from mature oligodendrocytes. The frequency of mature oligodendrocytes was significantly diminished (*t*_*6*_ = 12.66, *p* < 0.0001) in *Tuba1a-Cre::SELENOI*^*fl/fl*^ mice, and this corresponded with increased lipid peroxidation in both OPCs (OPCs: *t*_*6*_ = 17.69, *p* < 0.0001) and mature oligodendrocytes (Mature Oligos: *t*_*6*_ = 32.83, *p* < 0.0001) (Fig. 6, *C* and *D*). Interestingly, among cell types, levels of lipid peroxidation were highest in OPCs, which is consistent with prior findings that this cell type is particularly susceptible to oxidative stress (31) and that vulnerability decreases as maturation progresses (32, 33, 34). Additionally, to investigate whether SELENOI deficiency may impact other antioxidant systems, we measured brain levels of reduced, oxidized, and total glutathione and observed no differences between WT and *Tuba1a-Cre::SELENOI*^*fl/fl*^ mice (Fig. S8).

### SELENOI deficiency impedes myelination and OPC maturation *in vitro*

**Figure 7:**
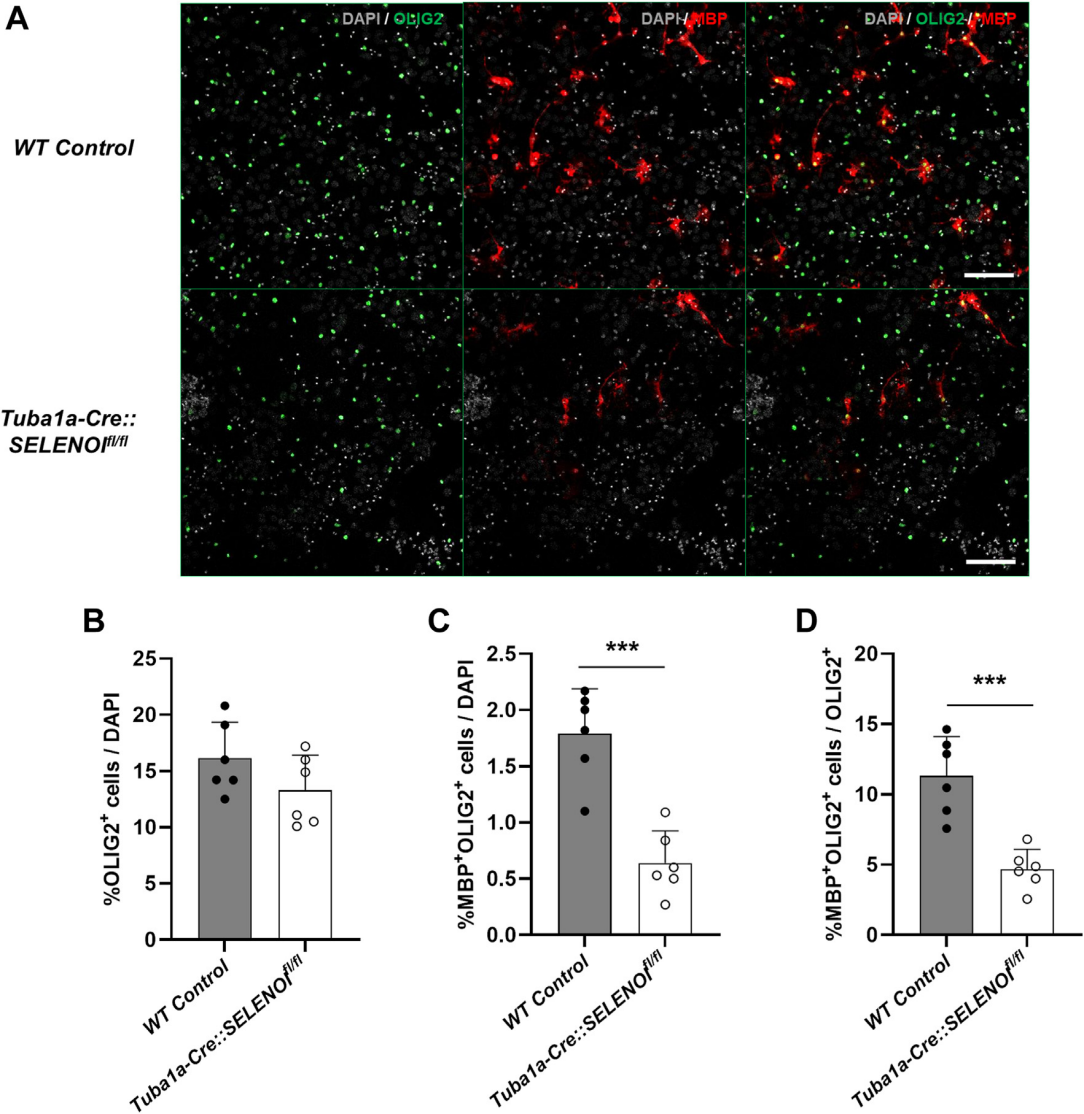
**SELENOI deficiency impedes myelination and OPC maturation *in vitro*.***A*, images of DIV21 primary cultures colabeled for OLIG2 (*green*) and MBP (*red*). *B* and *C*, density of OLIG2^+^ (*B*) and MBP^+^OLIG2^+^ (*C*) cells relative to DAPI. *D*, density of MBP^+^OLIG2^+^ relative to total OLIG2^+^ cells. N = 12 images from 12 separate coverslips per genotype. In all plots, *points* represent measurements from a single image and *bars* represent mean ± SD. The scale bar represents 100 μm; ∗∗∗*p* < 0.001. DAPI, 4′,6-diamidino-2-phenylindole; OPC, oligodendrocyte precursor cell; SELENOI, selenoprotein I; MBP, myelin basic protein.

To further probe the influence of SELENOI on oligodendrocyte development, we next performed *in vitro* experiments on mixed primary neuron/glia cocultures to corroborate our flow cytometry data. Cells derived from embryonic mouse cortex were cultured in Neurobasal media for 21 days, fixed, and immunolabeled with antibodies for MBP and the oligodendrocyte lineage marker, OLIG2 (Fig. 7*A*). Whereas the observed density of OLIG2-positive cells was comparable between genotypes (Fig. 7*B*), the proportion of mature, MBP-positive oligodendrocytes was significantly diminished in cultures derived from *Tuba1a-Cre::SELENOI*^*fl/fl*^ mice relative to both the total number of cells (*t*_*10*_ = 5.733, *p* = 0.0002) and the total number of oligodendrocyte lineage cells (*t*_*10*_ = 5.200, *p* = 0.0004) (Fig. 7, *C* and *D*).

## Discussion

Herein, we develop and characterize a mouse model of nervous system-restricted SELENOI deficiency that faithfully exhibits clinical elements of HSP displayed by humans with rare SELENOI mutations. These features include motor impairment, hypomyelination, microcephaly, and reduced body weight. Our results also show for the first time that SELENOI deficiency alters ether lipid composition within the brain, as abundance of ether-linked PE species were diminished and levels of ether-linked PC species were substantially elevated. Notably, similar changes in ether lipids have been reported in cell culture studies of HeLa cells and patient-derived fibroblasts lacking functional copies of SELENOI (11). Finally, the changes in ether lipid composition coincided with increased lipid peroxidation, demonstrating a protective role for SELENOI-derived plasmenyl-PE in neural cell types.

In accord with previous reports, we found that the absence of SELENOI leads to prominent changes in Kennedy pathway-derived lipid content (11, 35). Prior evidence indicates that the enzyme choline/ethanolamine phosphotransferase (CEPT1), which synthesizes both PE and PC, likely compensates when SELENOI is nonfunctional (35, 36, 37). Our lipidomics data (Figs. 5 and S7) show that the compensatory capacity of CEPT1 for diacyl lipids is relatively efficient. Specifically, as CEPT1 is diverted away from the choline branch of the Kennedy Pathway to maintain the ethanolamine branch, levels of both diacyl-PE and diacyl-PC are only slightly diminished proportionally. In contrast, when the lipid donor is AAG, the ability for CEPT1 to compensate for SELENOI is less efficient (35), as observed levels of ether-linked lipids were substantially perturbed in *Tuba1a-Cre::SELENOI*^*fl/fl*^ mice. This suggests that CEPT1 is less effective at carrying out a compensatory switch from choline to ethanolamine branches of the Kennedy Pathway when AAG serves as the lipid donor. Thus, lower plasmenyl-PE synthesis leads to heightened availability of AAG for incorporation into ether-linked PC species.

Strikingly, although concentrations of plasmenyl-PC species increased three-fold in *Tuba1a-Cre::SELENOI*^*fl/fl*^ mice, resulting in comparable levels of total plasmenyl species, this elevation proved insufficient to counteract lipid peroxidation and normalize myelination. Plasmenyl-PE content varies among tissues and cell types, with highest levels in brain white matter (38, 39). In contrast, plasmenyl-PC is most abundant in heart and minimally present in brain (40, 41). Moreover, due to their head group structure, PC species preferentially localize to the outer leaflet of membranes, while PE species prefer the inner leaflet (42, 43, 44, 45, 46). Given this asymmetric distribution, it is plausible that plasmenyl-PE deficiency increased lipid peroxidation in the inner leaflet and that plasmenyl-PC could not quench lipid peroxidation in this locale. Furthermore, aberrations in the ratio of PC to PE have been linked to disrupted membrane integrity (47) and compromised mitochondrial ATP production (48). Recent findings also indicate that plasmenyl-PE biosynthesis is spatiotemporally regulated by a feedback mechanism that senses levels in the inner leaflet and accordingly modulates the stability of fatty acyl-CoA reductase (FAR1), the rate-limiting enzyme of ether lipid synthesis within the peroxisome (46). This notion is supported by our data showing that plasmenyl-PE deficiency in *Tuba1a-Cre::SELENOI*^*fl/fl*^ mice corresponded with a two-fold increase in total ether lipids in the brain. It is important to note that the lipidomics data displayed in Figure 5 are normalized to total lipid content and does not correspond to actual measured values (Fig. S9), as the amount of total lipids were elevated by ∼64% in *Tuba1a-Cre::SELENOI*^*fl/fl*^ mice. This was largely due to augmented levels of PC species, leading to an increase in the ratio of total PC:PE. These alterations resulted in comparable measured levels of total PE between genotypes, with only plasmenyl-PE species being lower in *Tuba1a-Cre::SELENOI*^*fl/fl*^ mice.

Our observations add to a growing body of evidence implicating plasmenyl-PE as indispensable for proper neurodevelopment. In addition to SELENOI, mutations in the upstream enzyme that generates CDP-ethanolamine, CTP-phosphoethanolamine cytidylyltransferase (PCYT2), have been linked to HSP (49, 50, 51, 52). Furthermore, loss-of-function mutations in four genes (PEX7, GNPAT, AGPS, and FAR1) that mediate peroxisomal synthesis of AAG, a necessary substrate for synthesis of plasmenyl-PE and plasmenyl-PC, result in rhizomelic chondrodysplasia punctata (RCDP) (39, 53, 54, 55, 56, 57). RCDP is a developmental disorder that shares many features of HSP, including spasticity, intellectual impairment, retarded growth, microcephaly, and compromised myelination. Conversely, autosomal dominant mutations in FAR1 that promote unrestrained ether lipid synthesis lead to spastic paraplegia and delayed development without microcephaly and diminished growth (44). Of further relevance, HSP has also been linked to mutations of the calcium-independent phospholipase A2 beta (PLA2G6) (58, 59), a key enzyme influencing phospholipid metabolism, which acts by hydrolyzing fatty acyl groups at the sn-2 position, yielding lysophospholipids and free fatty acids (60, 61).

These findings also demonstrate that plasmenyl-PE is especially critical for developing oligodendrocytes. Among neural cells, concentrations of plasmenyl-PE are highest in oligodendrocytes, with levels roughly three-fold higher than neurons (38). Moreover, while lipid peroxidation was elevated in all neural cell types of *Tuba1a-Cre::SELENOI*^*fl/fl*^ mice, levels were highest in OPCs. Additionally, observed cell frequencies were comparable for all cell types except mature oligodendrocytes, suggesting that heightened lipid peroxidation in OPCs may serve as a barrier preventing maturation. Likewise, prior studies have demonstrated that OPCs are more susceptible to redox imbalance elicited by GSH depletion or exogenous reactive oxygen species than mature oligodendrocytes (33). Furthermore, this increased vulnerability appears to be due to low endogenous GSH content and high concentrations of iron, as comparative studies of OPCs and astrocytes found that iron levels were 20-fold higher in OPCs, while GSH content was 3-fold higher in astrocytes (62, 63). Notably, a later study reported similar GSH levels in astrocytes and mature oligodendrocytes (64), which suggests that GSH levels rise during oligodendrocyte maturation and thereby provide added protection. Recent studies have shown that plasmenyl-PE content in myelin increases three-fold during the rapid phase of myelination in mice (P15–P40), and this is accompanied by a decline in diacyl- and ether-linked PC species (28). Moreover, the elevation in plasmenyl-PE is preceded by oligodendrocyte-specific upregulation of SELENOI and upstream enzymes (ENTK1/2, PCYT2) of the ethanolamine branch of the Kennedy pathway, providing further corroboration for a consequential role of SELENOI-derived plasmenyl-PE in oligodendrocyte maturation.

**Figure 8:**
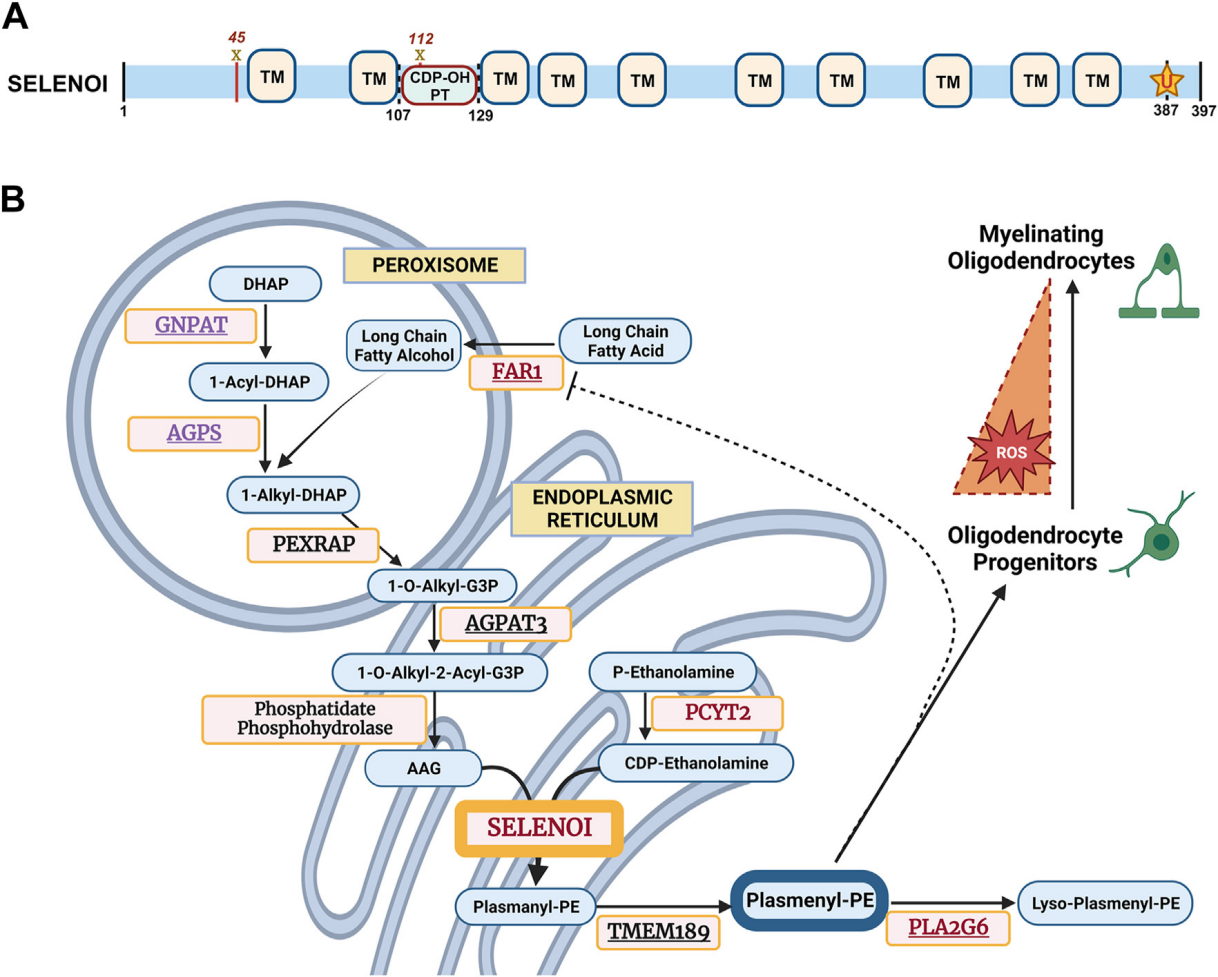
**Postulated influence of SELENOI-mediated plasmenyl-PE synthesis on oligodendrocyte maturation.***A*, domain organization of SELENOI. Note the CDP-OH PT domain spanning residues 107 to 129 and the presence of Sec (U) at residue 387. Mutations of residues 45 (Pro45Leu) (12) and 112 (Arg112Pro) (10) result in HSP in humans. *B*, biosynthesis pathway of plasmenyl-PE. Enzymes involved in plasmenyl-PE metabolism are designated by capital letters. *Red font* denotes enzymes with mutations linked to HSP (10, 11, 44, 49, 58). *Violet font* designates enzymes with mutations linked to RCDP (39, 53, 54, 77). *Underlined* enzymes correspond to those identified as key regulators of ferroptosis *via* genome-wide screening in cancer cell lines (74, 75) or using CRISPR/Cas9-engineered mutant mice (71). CDP-OH PT, CDP-ethanolamine phosphotransferase; HSP, hereditary spastic paraplegia; PE, phosphatidylethanolamine; RCDP, rhizomelic chondrodysplasia punctata; SELENOI, selenoprotein I.

Of additional significance, our results expand the known ensemble of individual selenoproteins with consequential roles within the nervous system. However, in contrast to other selenoproteins, the influence of SELENOI on redox balance appears to be mediated indirectly *via* plasmenyl-PE biosynthesis. Whereas selenocysteine (Sec) residues are typically situated within the catalytic domain of selenoproteins and serve to facilitate redox reactions, the Sec residue in SELENOI is not positioned within an established redox motif. In humans, SELENOI is comprised of 397 amino acids, including ten transmembrane domains, the catalytic domain for CDP-alcohol phosphotransferase (CDP-OH PT) activity, and the Sec residue in the C terminus (Fig. 8*A*). The conserved CDP-OH PT domain is also found in the CEPT1, which synthesizes both PE and PC. Given that CEPT1 is less efficient at PE synthesis relative to SELENOI (35, 37), the Sec residue may promote substrate specificity for CDP-ethanolamine, although this has yet to be established experimentally. Alternatively, the Sec residue may facilitate a yet to be determined redox reaction that is independent of its function as a CDP-OH PT.

Finally, several recent publications have reported that oligodendrocytes are vulnerable to ferroptosis (32, 65, 66, 67), an iron-dependent, nonapoptotic form of cell death characterized by elevated lipid peroxidation. Selenium protects against ferroptosis (68), as the selenoenzyme glutathione peroxidase 4 is essential due to its ability to reduce phospholipid hydroperoxides. Mice lacking glutathione peroxidase 4 in the nervous system succumb to fatal seizures shortly after birth due to ferroptosis-induced death of PVIs (27, 69). While we did not detect PVI loss in *Tuba1a-Cre::SELENOI*^*fl/fl*^ mice, it is conceivable that the reduction of mature oligodendrocytes resulted, in part, from ferroptosis of OPCs. Although SELENOI has yet to be directly linked, PE metabolism is a pivotal regulator of this process, as oxidized arachidonic (20:4) and adrenic (22:4) fatty acid chains in diacyl-PE act as potent ferroptotic death signals (70, 71, 72). Likewise, due to its ability to hydrolyze oxygenated diacyl-PE species that promote ferroptotic death, the enzyme PLA2G6 provides vital protection against ferroptosis (71, 73). Additional data generated from genome-wide screens of cancer cell lines identified multiple genes involved in ether lipid synthesis (AGPAT3, AGPS, FAR1, GNPAT, and TMEM189) as critical contributors to ferroptosis vulnerability, findings further validated by gene silencing experiments (74, 75). Intriguingly, as noted above, mutations in GNPAT, AGPS, and FAR1 have been linked to RCDP (53, 54, 56), while certain mutations in PLA2G6 and FAR1 cause HSP (44, 58, 59) (Fig. 8*B*). Taken together, these observations suggest that defects in plasmenyl-PE biosynthesis may disproportionately increase vulnerability of developing oligodendrocytes to ferroptosis, and that ensuing disruption of oligodendrocyte maturation may be the root cause of the hypomyelination, microcephaly, and spasticity that is characteristic of both HSP and RCDP.

## Experimental procedures

### Mice

The generation and genotyping of *SELENOI*^*fl/fl*^ mice have been previously described (14), and *Tuba1a-Cre* mice were a generous gift of Dr Lino Tessarollo (17). These mice were mated to generate *Tuba1a-Cre::SELENOI*^*fl/fl*^ mice. In addition, we bred *Tuba1a-Cre* mice with *ROSA26R*^**tdTomato**^ reporter mice (76) to profile Cre recombinase expression. For all experiments, sex- and age-matched *SELENOI*^*fl/fl*^ littermates were used as WT controls. All procedures and experimental protocols were approved by the University of Hawaii’s Institutional Animal Care and Use Committee and conducted in accordance with the Association for Assessment and Accreditation of Laboratory Animal Care and National Institutes of Health guidelines. Behavioral studies were performed on young adult mice at 8 to 12 weeks of age, in the following order: (1) open field test, (2) Rotarod, and (3) vertical pole descent, with each test separated by several days. Experimental groups consisted of equal numbers of males and females. For all studies, male and female *Tuba1a-Cre::SELENOI*^*fl/fl*^ mice showed similar trends relative to sex-specific WT controls. Thus, males and females were pooled for graphical presentation and statistical analyses in the main figures, unless otherwise noted. Data disaggregated by sex are displayed in the Supplementary Figures.

### Histology and immunohistochemistry

Mice were deeply anesthetized (1.2% avertin; 0.7 ml/mouse) and perfused intracardially with cold 0.1 M phosphate buffer (PB) followed by 4% paraformaldehyde (PFA) in PB. Brains were removed, stored in 4% PFA for 24 h, immersed in graded solutions of sucrose (10%, 20%, and 30%), and cut into 40 μm coronal sections. For immunohistochemistry, free-floating sections were treated with 0.3% H_2_O_2_ to inactivate endogenous peroxidases, blocked, and incubated overnight at 4 °C with proper primary antibodies. The next day, sections were probed with the appropriate biotinylated secondary antibody followed by incubation in avidin-biotin-peroxidase complex (ABC Elite Kit, Vector Labs), and immunoreactivity was visualized by peroxidase detection using diaminobenzidine tetrahydrochloride (DAB Substrate Kit, Vector Labs) as a chromogen substrate. After several rinses in PBS, sections were mounted on slides, dehydrated with graded solutions of EtOH followed by xylene, and coverslipped.

### Antibodies

The following antibodies were used for Western blot at their indicated dilutions: anti-EPT1 (Elabscience, Cat# E-AB-40549, 1:1000) and anti-vinculin (Santa Cruz Biotechnology, RRID:AB_131294; 1:1000); fluorescent secondary antibodies specific to primary antibody host species were used for visualization on (LI-COR Biosciences). The following antibodies were used for immunohistochemistry at their indicated dilutions: anti-GFAP (Sigma-Aldrich, RRID:AB_477010, 1:500), anti-MBP (Cell Signaling, RRID:AB_2799920, 1:500), and anti-OLIG2 (EMD Millipore, RRID:AB_10907410, 1:500).The following antibodies were used for flow cytometry at their indicated dilutions: anti-ACSA-2-biotin (Miltenyi Biotech, RRID:AB_2904626, 1:50), anti-CD11b-biotin (Miltenyi Biotech, RRID:AB_2726319, 1:50), anti-Olig4-biotin (Miltenyi Biotech, RRID:AB_2751959, 1:50), anti-MOG-APC (Miltenyi Biotech, RRID:AB_2905334, 1:50), and anti-mouse CD16/32 (Fc Block) (BioLegend, RRID:AB_312801; 1:50).

### BaseScope *in situ* hybridization

Mice were perfused with 0.1 M PB followed by 10% formalin for use in BaseScope studies. Following fixation for 24 h in 10% formalin, brains were dehydrated, washed in xylene, and embedded in paraffin using standard procedures. Formalin-fixed, paraffin-embedded tissue was sectioned at 5 μm thickness and mounted onto SuperFrost slides. BaseScope *in situ* hybridization (Advanced Cell Diagnostics) was conducted according to the manufacturer’s guidelines. We utilized a custom designed probe for detection of Selenoi, along with positive (Ppib) and negative (DapB) control probes supplied by the manufacturer. In brief, tissue sections were deparaffinized, treated with H_2_O_2_, and incubated in 1× target retrieval reagent for 15 min at 99 °C in a steamer. Tissue was then permeabilized by applying RNAscope Protease IV for 30 min at 40 °C. Next, BaseScope probes were hybridized to tissue for 2 h at 40 °C. Following probe amplification steps, transcripts were detected with the BaseScope RED detection kit. Sections were then counterstained with hematoxylin, cleared with xylene, and coverslipped.

### Quantitative PCR

Measurement of *Selenoi* mRNA levels in brain and skeletal muscle were conducted as described previously (11). Ubiquitin C was used as a reference/housekeeping gene.

### Open field test

Mice were placed in the center of an open field apparatus (50 × 50 cm) protected with 10 cm high walls and allowed to explore for 5 min. Locomotion was recorded by a video camera connected to a PC and analyzed by video tracking software (VideoMot2, TSE Systems).

### Rotarod test

Starting speed for the Rotarod was 4 rpm and increased to 40 rpm over a 5 min period. The latency to fall off the rod was measured for each trial. Mice received four trials of training, with the best score used for statistical analysis.

### Vertical pole descent test

Mice were placed head-up on top of a vertical wooden pole (length = 50 cm; diameter = 1.2 cm) with a plexiglass base that was inserted into the animal’s home cage. When placed on the pole, mice orient themselves downward and descend the length of the pole back into their home cage. Mice received four trials of training a day for two consecutive days, with the latency to orient downward and descend the length of the pole recorded for each trial. Best scores were used for statistical analyses.

### Electron microscopy

Adult mice (15 weeks) were perfused with 0.1 M PB followed by primary fixative solution (2% paraformaldehyde + 2.5% glutaraldehyde in 0.2 M sodium cacodylate buffer). Brains were collected, sectioned into 1 mm thick coronal sections, stored in primary fixative for 24 h at 4 °C, and further dissected to isolate regions for embedding. Likewise, sciatic nerves were also collected and stored in primary fixative for 24 h at 4 °C. Isolated tissues were then washed 2 × 30 min in 0.1 M sodium cacodylate buffer. Subsequently, tissues were postfixed in 2% osmium tetroxide in 0.1 M sodium cacodylate buffer for 1 h, followed by 3 × 10 min incubations in propylene oxide. Next, tissue was infiltrated overnight with 1:1 propylene oxide:LX-112 resin. LX-112 resin consists of 2.3 g LX-112 (Ladd Research, Cat# #21310), 1.02 g Dodecenyl Succinic Anhydride (Ladd Research, Cat#21340), 1.28 g Nadic Methyl Anhydride (Ladd Research, Cat# 21350), and 115 ml N-Benzyldimethylamine (Ted Pella, Cat# 18241). Brain tissues were then incubated with freshly made LX-112 resin for 2 h and 4 h, changing resin in between incubations. Tissue was then placed in silicon molds, covered in LX-112 resin, and incubated at 60 °C for 72 h to polymerize resin. Sections were cut at a thickness of 900 nm and images were obtained using a Hitachi 7700 Transmission Electron Microscope. Quantification of myelination in individual neurons was determined by the myelin g-ratio, which is defined as the ratio of the inner (axon) to the outer (axon + myelin) diameter of the nerve fiber.

### Silver staining

Silver staining was performed using the FD Neurosilver Kit II (FD NeuroTechnologies, Inc) according to the manufacturer’s instructions.

### GSH measurement

Levels of reduced, oxidized, and total glutathione were measured using a GSH/GSSG ratio detection assay kit (Abcam) according to the manufacturer’s instructions. Fluorescence was measured on a Molecular Devices SpectraMax M3 microplate reader.

### Lipidomics sample preparation

Brain samples were weighed and homogenized using a Bertin Precellys 24 Tissue Homogenizer in a screw-top microcentrifuge tube containing ∼0.1 ml of zirconia/silica beads (1 mm, BioSpec Products) and 1.0 ml of LC-MS grade isopropanol:water:ethyl acetate (30:10:60, *v:v:v*) containing SPLASH Lipidomix Mass Spec Standard (Avanti Polar Lipids). This was followed by sonication for 5 min and then centrifugation for 5 min at 15,000*g* at 4 °C. Supernatants were transferred to new tubes and brought to dryness using a Thermo Savant SPD121P Speedvac Concentrator. They were then reconstituted in 0.5 ml of LC-MS grade isopropanol:acetonitrile:water (45:35:20, *v:v:v*), vortexed briefly, and sonicated for 5 min. Finally, samples were centrifuged for 10 min at 15,000*g* at 4 °C and supernatant was transferred to auto-sampler vials.

### LC-MS/MS parameters for lipidomics

Samples were acquired on a Thermo Orbitrap Fusion Lumos Tribrid mass spectrometer coupled with a Thermo Vanquish ultra high performance liquid chromatography system and a Waters CSH C18 column (1.0 × 150 mm × 1.7 μm particle size). Solvent A was LC-MS grade acetonitrile:water (60:40, *v:v*) containing 10 mM ammonium formate and 0.1% formic acid and solvent B was LC-MS grade isopropanol:acetonitrile (95:5, *v:v*) containing 10 mM ammonium formate and 0.1% formic acid. The mobile phase flow rate was 0.11 ml/min with column temperature at 65 °C. The gradient of solvent B was as follows, 0 min 15% (B), 0 to 2 min 30% (B), 2 to 2.5 min 48% (B), 2.5 to 11 min 82% (B), 11 to 11.01 min 99% (B), 11.01 to 12.95 min 99% (B), 12.95 to 13 min 15% (B), and 13 to 15 min 15% (B). Ion source spray voltages were 4000 V and 3000 V in positive and negative mode, respectively. The mass spectrometry collected full scan data from 200 to 1600 m/z and utilized AcquireX mode with stepped collision energies of 30% ± 5% and 40% ± 20% for collecting tandem mass spectrometry scans in positive and negative mode, respectively. A pooled quality control sample and standards mix quality assurance sample were used to ensure analytical reproducibility and sensitivity. Measured data were normalized as nmol lipid species per milligram brain tissue. Data displayed in Figure 5 are represented as the percentage of a given lipid class relative to the total lipid content.

### Whole brain single-cell suspension

Three-week-old mice were sacrificed by CO_2_ asphyxiation and brains were collected in cold Hank’s buffered salt solution (HBSS). All centrifugation steps were done at 350*g* for 5 min at 4 °C. Brains were minced in HBSS, centrifuged, and the pellet was resuspended in 1.2 ml of digestion buffer (Neuronal Isolation Enzyme with Papain, Thermo Fisher Scientific; Cat# 88285) for 30 min at 37 °C and 5% CO_2_. After incubation, HBSS was added to 50 ml, centrifuged, and the supernatant was discarded. With a 10 ml serological pipette, 10 ml of serum-free culture medium consisting of Neurobasal Plus Medium (Thermo Fisher Scientific, Cat# A3582901), 2% B27 Supplement minus antioxidants (Thermo Fisher Scientific, Cat# 10889038), 1% GlutaMAX Supplement (Thermo Fisher Scientific, 35050061), 1% Antibiotic-Antimycotic (Thermo Fisher Scientific, 15240062) was added to the pellet, and the brain matter was triturated. Volume was raised to 50 ml with HBSS, centrifuged, and the brain was triturated with 2 ml of serum-free culture medium and a P1000 pipette tip until single cell suspension was achieved. Volume was then brought up to 50 ml with HBSS, centrifuged, and pellet was then resuspended in 2 ml of serum-free culture medium. Volume was raised to 25 ml with HBSS, and cell suspension was filtered through a 100 μm filter. Filter was then washed with 25 ml HBSS. Cell suspension was centrifuged, pellet was resuspended in 10 ml serum-free culture medium, and cell count and viability were assessed.

### Flow cytometry

Single cell suspensions at a concentration of 10^6^ cells/ml were incubated with 1 μM C11-BODIPY 581/591 (Thermo Fisher Scientific, Cat# D3861) for 30 min at 37 °C and 5% CO_2_. Cells were then centrifuged and resuspended in staining buffer (2% fetal bovine serum in PBS) at a concentration of 10^7^ cells/ml. Two million cells (0.2 ml) were stained in quadruplicate for each staining condition. Cells were seeded in a 96-well plate, centrifuged, and resuspended in Fc Block in staining buffer for 10 min at 4 °C. The cells were then incubated for 30 min at 4 °C with appropriate antibodies diluted in staining buffer to achieve the final concentrations stated above, as well as LIVE/DEAD Fixable Violet Dead Cell Stain (Invitrogen, Cat# L34955) following manufacturer's recommendation. Cells were washed twice with staining buffer and stained for 30 min at 4 °C with PE/Cy7 Conjugated Streptavidin (eBiosciences, Cat# 25-4317-82) in staining buffer (1:1000). Cells were then washed twice with staining buffer, fixed in Fixation Buffer (BioLegend, Cat# 420801) for 15 min at room temperature, and then resuspended in staining buffer. Flow cytometry was subsequently performed using a BD LSRFortessa cell analyzer, and the data were analyzed using FlowJo software (www.flowjo.com).

### Primary neuron/glia cocultures

Primary cultures were derived from cortices of E16 *Tuba1a-Cre::SELENOI*^*fl/fl*^ and *SELENOI*^*fl/fl*^ littermate control mice. Following trituration, isolated cells were plated on poly-D-lysine-coated coverslips/wells (150,000 cells/cm^2^) and grown in neurobasal media at 37 °C and 5% CO_2_, with half-volume media exchanges performed biweekly. At DIV21, cultures were fixed in 4% PFA for 15 min and subsequently labeled with antibodies against OLIG2 and MBP to investigate oligodendrocyte maturation.

### Image analysis

Images were captured using bright-field (Zeiss Axioskop2) and confocal (Leica SP8) microscopes in the JABSOM Imaging Core and subsequently imported into FIJI (https://imagej.net/software/fiji/) and/or QuPath (https://qupath.github.io/) image analysis software. To quantify levels of immunoreactivity/staining in specific brain regions, contours were drawn around each region of interest (ROI) with the aid of a mouse brain atlas. In parallel, another contour was drawn around an area devoid of positive staining that served as a background control region. Mean absorbance for each ROI was determined by subtracting the absorbance of the background control region from the optical density of each ROI. These values were then normalized relative to the WT average. For measurements of cell density, images were first segmented using the Triangle threshold method in FIJI. Cell density was then determined using the “Analyze Particles” feature in FIJI with parameters set at 25 to 500 μm^2^ for size and 0.3 to 1.0 for circularity. To quantify the density of oligodendrocytes in primary culture, 4 × 4 tile scan images using a 20× objective were taken for the region containing the most robust MBP staining on each coverslip. Images were then imported into QuPath image analysis software, and numbers of oligodendrocyte lineage cells (OLIG2^+^) and mature oligodendrocytes (MBP^+^OLIG2^+^) were determined semiautomatically using the cell classifier feature. Raw numbers were normalized relative to total 4',6-diamidino-2-phenylindole cells for each image.

### Statistical analyses

Data were analyzed and plotted using GraphPad Prism software 10.1 (www.graphpad.com). For all figures, results are represented as mean ± SD and levels of significance are denoted as follows: ∗ for *p* < 0.05, ∗∗ for *p* < 0.01, ∗∗∗ for *p* < 0.001. Student’s *t* tests were used for parametric, two-group comparisons and calculated *p*-values for each comparison are indicated in the text and/or figure legends.

### Sample size

Determination of sample size was guided by ethical principles in the use of animals and aimed to minimize animal usage. Based upon our prior experience, we estimated that a minimum of six animals per group (three males, three females) would be needed for behavioral (Fig. 2) and histological (Figs. 3 and 4) studies to assess differences between genotypes and, if observed, to determine whether they were sex-specific. Given the consistent, robust differences we observed between genotypes and the fact that effects were not influenced by sex, a sample size of N = 4 (two males, two females) was used for lipidomic (Fig. 5) and flow cytometry (Fig. 6) studies of tissue/cells derived from 3 week-old mouse pups. For studies in primary culture (Fig. 6), dissected cortices from multiple embryonic pups from each genotype (N > 3) were pooled and plated on cover slips. Subsequently, 4 × 4 tile scan images were taken for the region containing the most robust MBP staining on each coverslip (N = 6) and images were analyzed using QuPath software.

## Data availability

All data that supports the findings of this study are available from the corresponding author upon request.

## Supporting information

This article contains supporting information.

## Conflict of interest

The authors declare that they have no conflicts of interest with the contents of this article.
