# Supplementary material for: Selenoprotein I is indispensable for ether lipid homeostasis and proper myelination
Source: J Biol Chem. 2024 Apr 4;300(5):107259. doi: 10.1016/j.jbc.2024.107259 (PMC11061234; doi:10.1016/j.jbc.2024.107259)
Supplement: Supporting Figures [file mmc2.pdf]

Selenoprotein I is indispensable for ether lipid homeostasis and proper myelination

Lance G.A. Nunes[1], Chi Ma[2], FuKun W. Hoffmann[2], Ashley E. Shay[3,4], Matthew W. Pitts[2,5], and Peter R. Hoffmann[2,5]

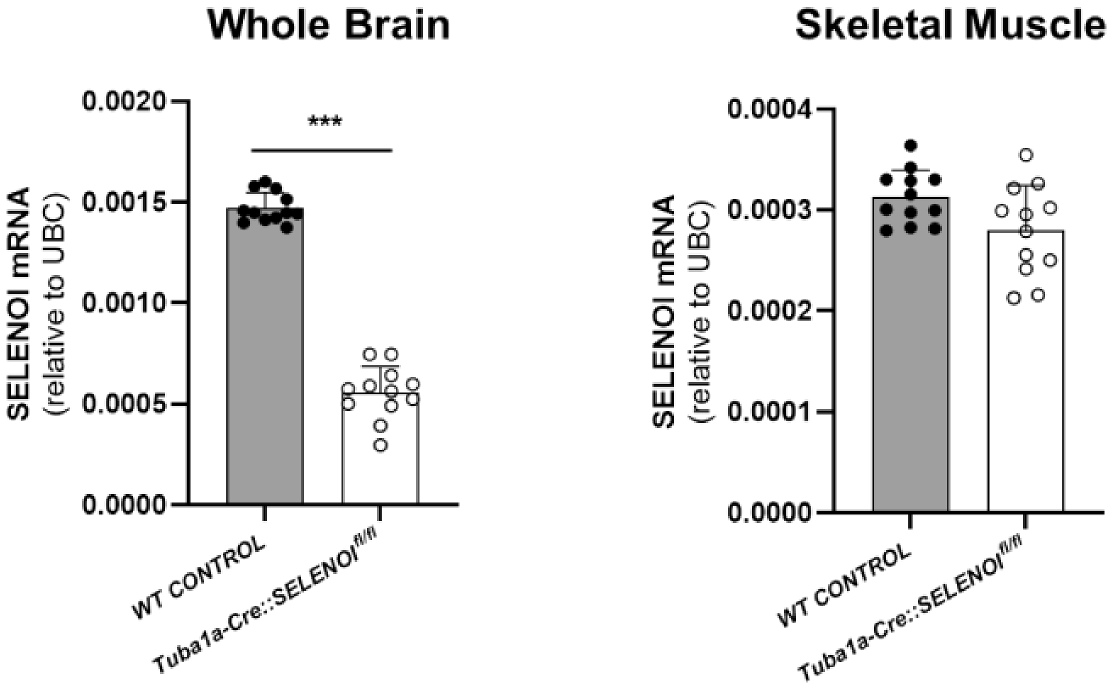

Figure S1. Measurement of SELENOI mRNA levels in Whole Brain and Skeletal Muscle. Measured levels of SELENOI mRNA in whole brain (left) and skeletal muscle (right) samples derived from WT Control and *Tuba1a-Cre::SELENOI* $^{fl/fl}$ mice. N = 12 (4 mice per genotype; 3 replicates per mouse). ****p*<0.001. Abbreviations: UBC, Ubiquitin C.

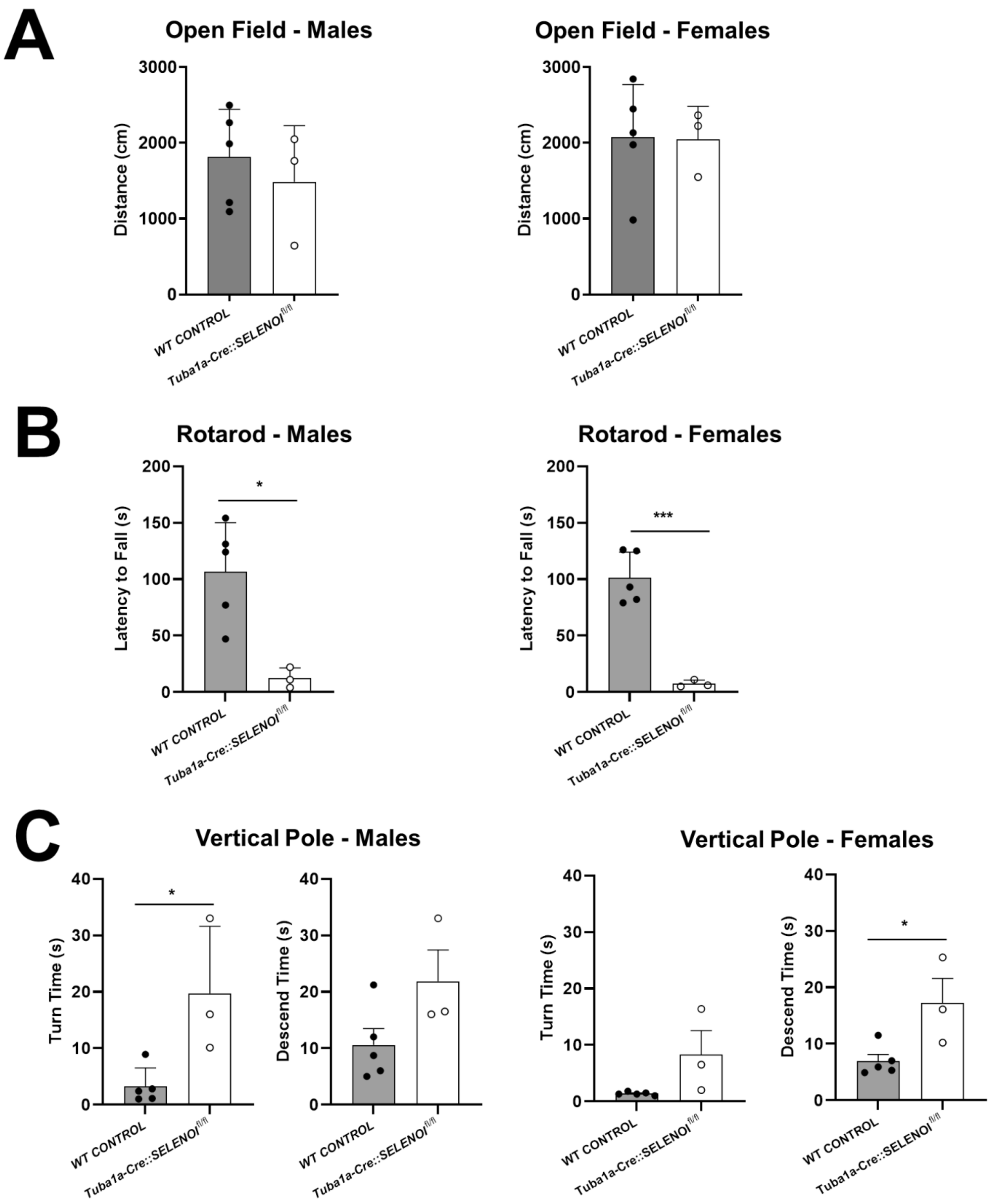

Figure S2. Characterization of *Tuba1a-Cre::SELENOI* $^{fl/fl}$ mice. (A) Distance travelled in the open field test for males and females. **(B)** Latency to fall off the Rotarod for males and females. **(C)** Time to turn downward and descend the vertical pole for males and females. N = 3 - 5 mice per genotype. **p*<0.05, ***p*<0.01, ****p*<0.001.

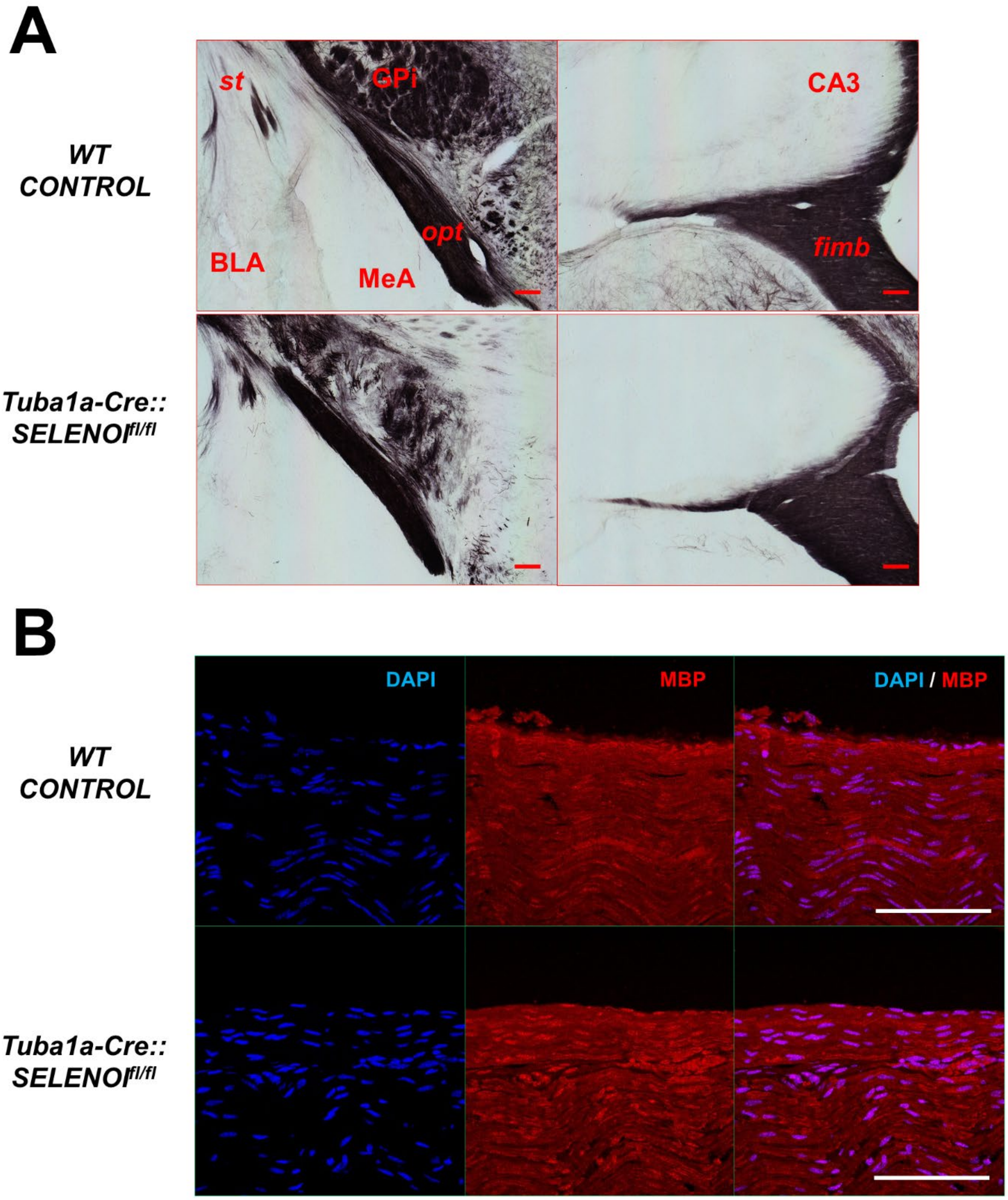

Figure S3. Comparable expression of myelin basic protein in non-motor CNS tracts and sciatic nerve of PNS. (A) Images of MBP immunostaining in the optic tract (left) and fimbria (right). (B) Images of MBP immunostaining in sciatic nerves. Scale bar = 100 μm. Abbreviations: BLA, basolateral nucleus of amygdala; CA3, CA3 region of hippocampus; fimb, fimbria; GPi, globus pallidus; MeA, medial nucleus of amygdala; opt, optic tract; st, stria terminalis.

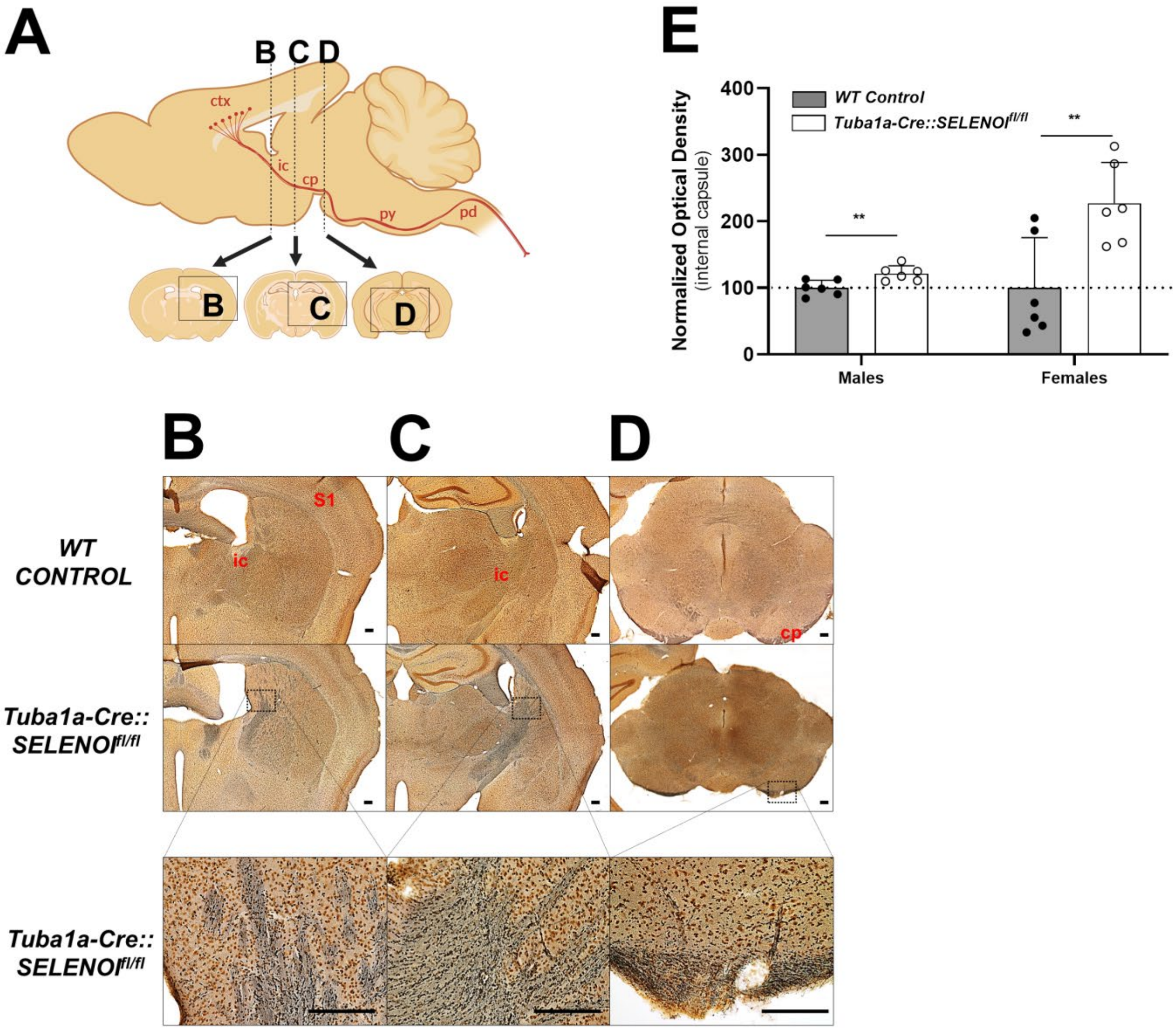

Figure S4. Neurodegeneration in the corticospinal tract of *Tuba1a-Cre::SELENOI* $^{fl/fl}$ mice. **(A)** Sagittal diagram of the mouse brain detailing the corticospinal tract in red. Dotted lines correspond to coronal sections used for image analysis. **(B - D)** Images of silver stained coronal sections containing the *ic* **(B, C)**, and *cp* **(D)**. **(E)** Quantification of optical density of silver staining in the *ic*. Scale bar = 100 µm. ***p*<0.01.

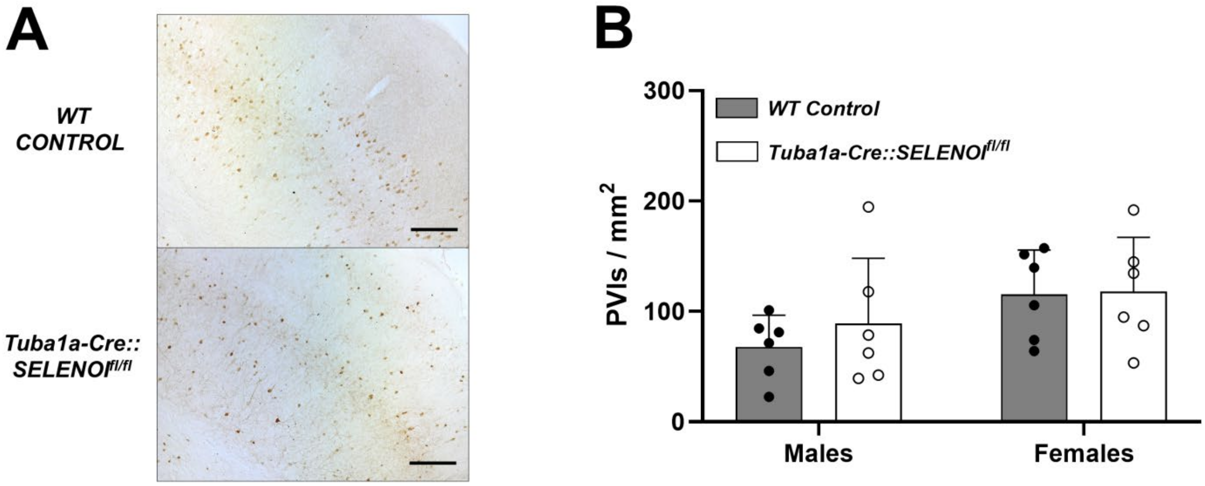

Figure S5. Comparable density of cortical parvalbumin-expressing interneurons in *Tuba1a-Cre::SELENOI* $^{fl/fl}$ mice. **(A)** Images of coronal sections immunostained for parvalbumin in S1 region of the cortex. **(B)** Quantification of the density of parvalbumin-expressing interneurons (PVIs) in S1. Scale bar = 100 µm.

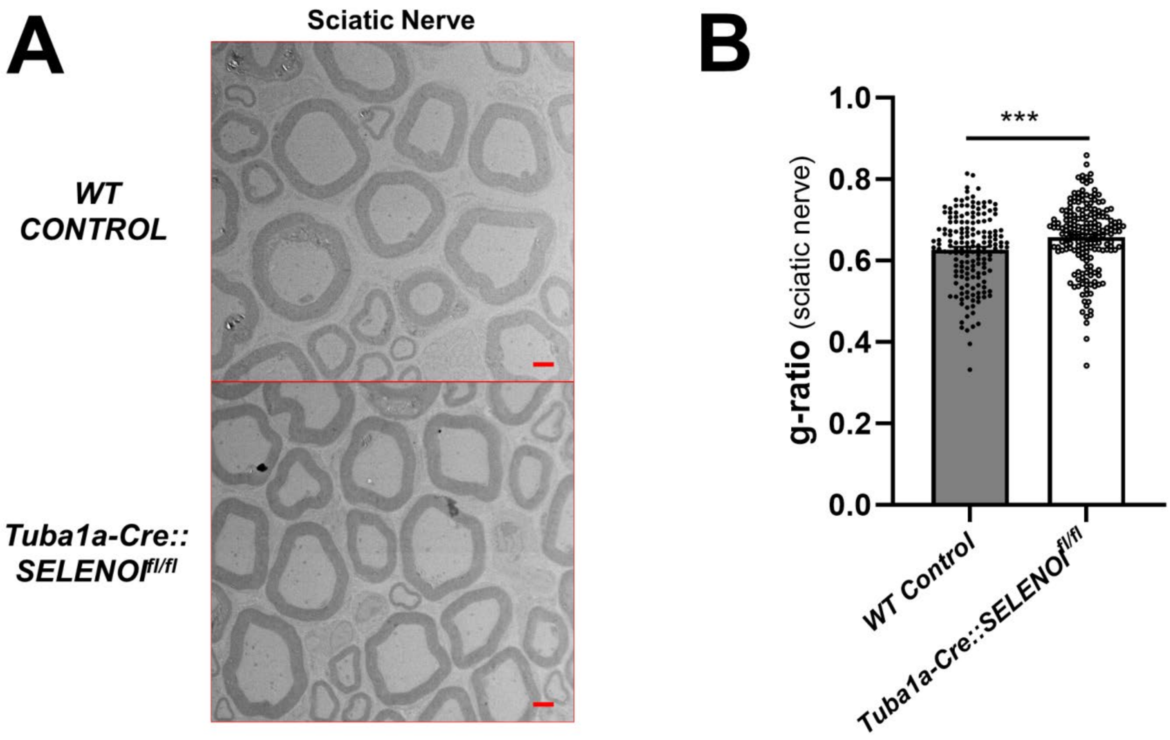

Figure S6. Subtle alterations in peripheral nervous system myelination of *Tuba1a-Cre::SELENOI*$^{fl/fl}$ mice. (A) Transmission electron microscopy images of myelin in sciatic nerves of the peripheral nervous system. **(B)** Quantification of myelin by determination of the g-ratio. In all plots, points represent measurements from a single image. Scale bar = 2 μm. ***p<0.001.

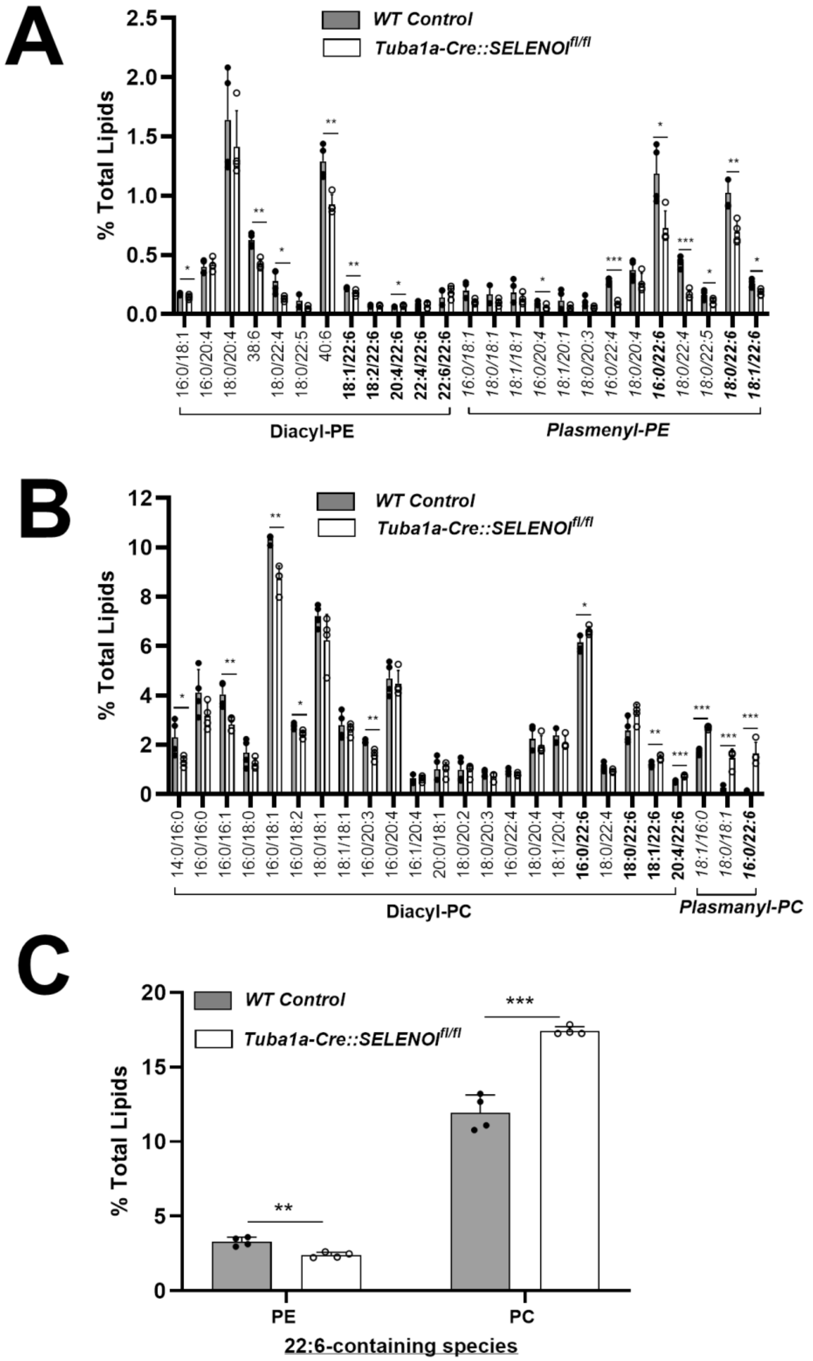

Figure S7. Comparative analyses of ethanolamine and choline phospholipid species in brains of *Tuba1a-Cre::SELENOI* $^{fl/fl}$ mice. (A) Relative levels of the 25 most abundant PE species. **(B)** Relative levels of the 25 most abundant PC species. **(C)** Relative levels of PE and PC species containing a fatty acid in the 22:6 configuration. Note that 22:6-containing lipid species are denoted by bold face text in A and B. N = 4 brains per genotype. **p*<0.05, ***p*<0.01, ****p*<0.001.

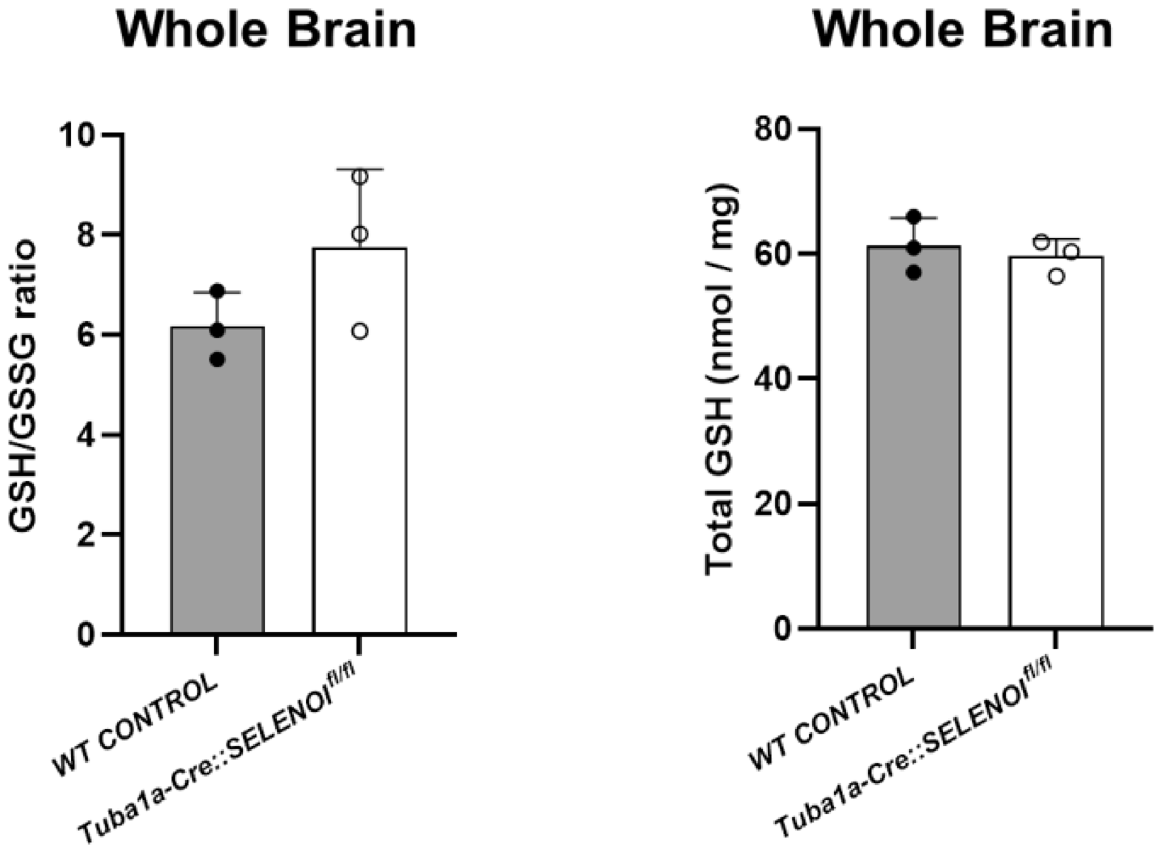

Figure S8. Glutathione Redox System is not altered in brains of *Tuba1a-Cre::SELENOI* $^{fl/fl}$ mice. The ratio of reduced (GSH) to oxidized (GSSG) glutathione (left) was comparable between WT Control and *Tuba1a-Cre::SELENOI* $^{fl/fl}$ mice. Likewise, similar levels of total glutathione were observed. N = 3 brains per genotype.

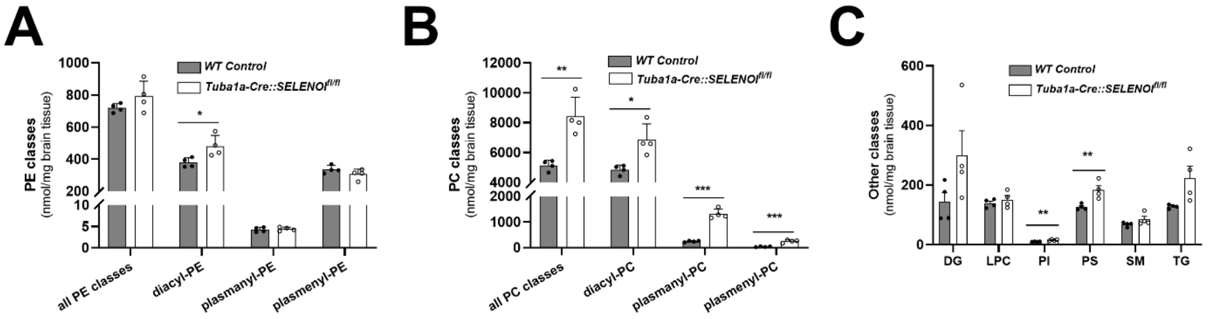

Figure S9. Measured levels of phospholipid species in brains of *Tuba1a-Cre::SELENOI* $^{fl/fl}$ mice. (A) Measured levels of PE lipid classes. **(B)** Measured levels of PC lipid classes. **(C)** Measured levels of major lipid classes synthesized outside of the Kennedy pathway. N = 4 brains per genotype. *p<0.05, **p<0.01, ***p<0.001.
